# Semiconducting Nanomaterials for Intrinsically Stretchable Field‐Effect Transistors

**DOI:** 10.1002/advs.202509642

**Published:** 2025-08-28

**Authors:** Seongmin Heo, Gwon Byeon, Soonhyo Kim, Taoyu Zou, Yong‐Young Noh

**Affiliations:** ^1^ Department of Chemical Engineering Pohang University of Science and Technology (POSTECH) Pohang Gyeongbuk 37673 Republic of Korea

**Keywords:** field‐effect transistors, intrinsically stretchable electronics, nanomaterials, semiconducting materials, wearable electronics

## Abstract

Developing intrinsically stretchable field‐effect transistors (FETs) is critical for enabling next‐generation flexible, wearable, and bio‐integrated electronic systems. Unlike conventional stretchable devices that rely primarily on geometric engineering of rigid materials, intrinsically stretchable FETs involve materials that inherently withstand large mechanical deformation while preserving their electronic performance. Although significant progress is achieved in the field of stretchable devices, further innovation in semiconductor materials and compatible process technologies remains essential for advancing the field. This review summarizes recent progress and challenges in intrinsically stretchable semiconducting nanomaterials. Various fabricating processes for stretchable devices are presented, together with recent applications of intrinsically stretchable FETs in sensory technologies, stretchable displays, digital computing, and biomimetic systems. Finally, the remaining challenges and perspectives are summarized for future research directions to realize highly scalable, durable, and high‐performance intrinsically stretchable FETs for next‐generation electronic platforms.

## Graphical abstract

This review discusses intrinsically stretchable field‐effect transistors, highlighting recent developments in stretchable semiconducting nanomaterials and fabrication methods. Key material strategies and innovative deposition techniques enabling enhanced mechanical elasticity alongside reliable electronic properties are covered. Additionally, emerging applications in wearable sensors, stretchable displays, biomimetic systems, and stretchable digital computing devices are presented, along with future research perspectives.

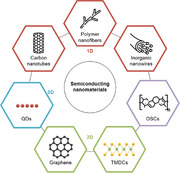

## 1 Introduction

The demand for wearable electronic devices such as smartwatches, smart clothes, and patches has increased recently, thus promoting the rapid development of medical systems that can monitor health remotely by detecting physical signals.^[^ ^1^, ^2^ ^]^ Such advancements have enabled more personalized healthcare and real‐time tracking of vital signs with minimal user intervention. However, most commercial wearable electronic devices are still rigid and bulky, making it difficult to adapt to the soft and flexible characteristics of the human body. This mismatch between the mechanical properties of traditional electronics and biological tissues limits comfort and affects the long‐term wearability and functionality of such devices.^[^ ^3^ ^]^ To overcome these challenges, electronic skin (E‐skin), a device that is extremely thin, soft, stretchable, and completely adherent to the human body, has emerged as a promising solution.

**Figure 1:**
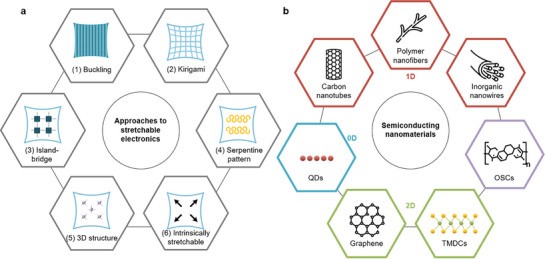
Schematic of the different approaches to afford stretchable electronics by a) structure engineering and b) material engineering in semiconducting nanomaterials.

Early studies on E‐skin focused on structural engineering with conventional rigid materials (**Figure** **1**a), involving notable methods such as: 1) Buckling involves the deposition of a stiff film on a pre‐strained substrate, forming a wavy structure upon release and allowing the material to stretch without mechanical failure.^[^ ^4^, ^5^, ^6^ ^]^ 2) The Kirigami method, inspired by traditional paper‐cutting techniques, introduces strategic cuts into the material to enable controlled stretching and deformation, enhancing both mechanical flexibility and robustness.^[^ ^7^, ^8^ ^]^ 3) Island‐bridge structures consist of rigid islands housing the active electronic components connected by flexible bridges that provide the necessary stretchability while maintaining functionality.^[^ ^9^, ^10^ ^]^ 4) Serpentine patterns involve wavy, serpentine configurations of conductive traces that allow for stretching and compression, integrating stretchable interconnects into flexible devices without compromising electrical integrity.^[^ ^11^, ^12^ ^]^ 5) 3D architectures, such as helical springs or interlocking networks, inherently provide stretchability by deforming in multiple directions, enhancing flexibility and durability under mechanical strain.^[^ ^13^, ^14^, ^15^ ^]^ Despite advancements in the performance of stretchable electronics based on geometric engineering strategies, significant challenges remain, including the complexity of the manufacturing process, low device integration density, high cost, and time consumption.^[^ ^16^, ^17^ ^]^ 6) Intrinsically stretchable electronics made entirely of inherently stretchable materials have emerged as a promising alternative to address these limitations.^[^ ^18^, ^19^, ^20^ ^]^

**Figure 2:**
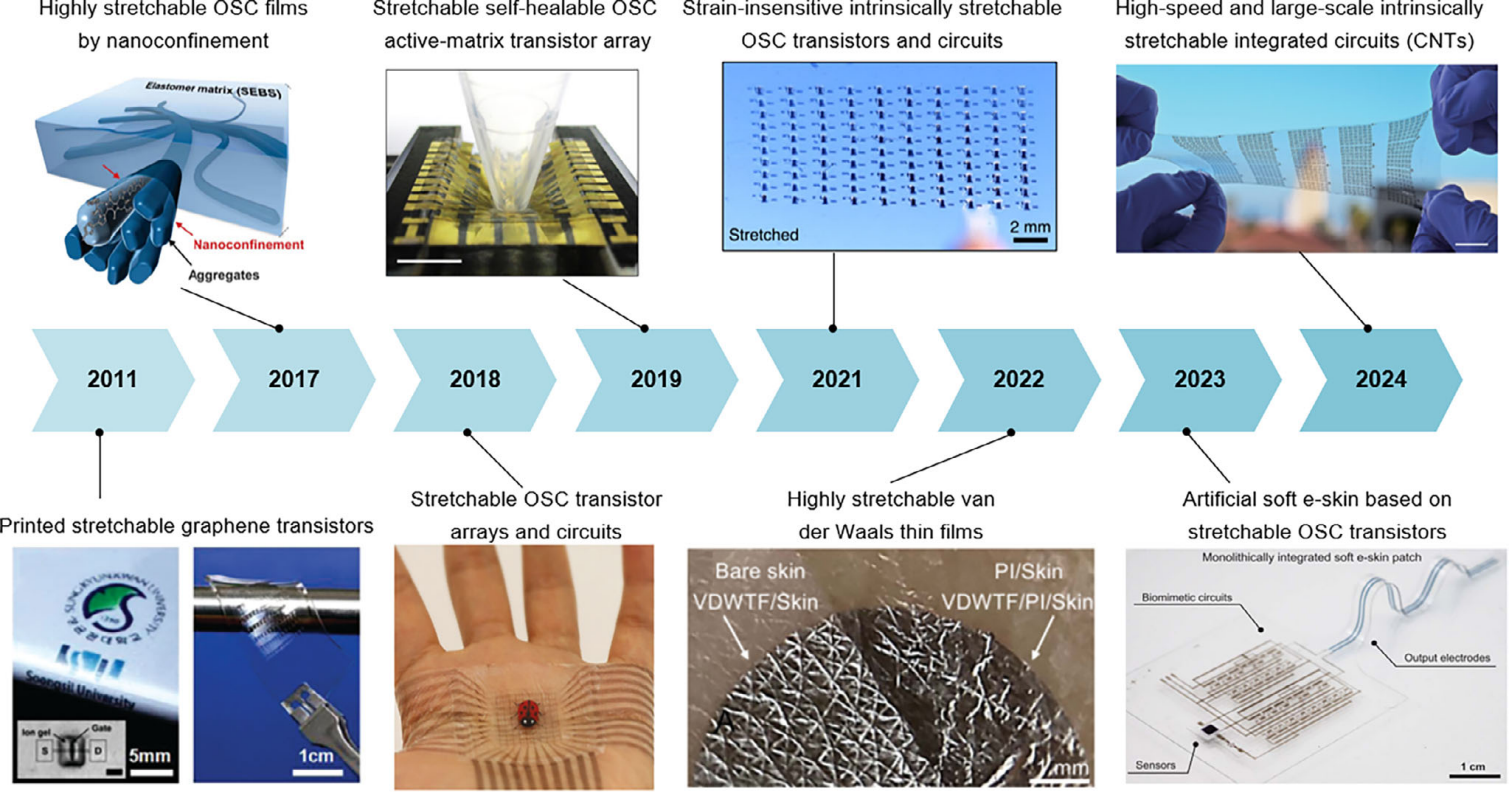
The development of intrinsically stretchable transistors. Reproduced with permission.^[^ ^26^ ^]^ Copyright 2011, American Chemical Society. Reproduced with permission.^[^ ^27^ ^]^ Copyright 2017, AAAS. Reproduced with permission.^[^ ^28^ ^]^ Copyright 2018, Springer Nature. Reproduced with permission.^[^ ^29^ ^]^ Copyright 2019, AAAS. Reproduced with permission.^[^ ^30^ ^]^ Copyright 2021, Springer Nature. Reproduced with permission.^[^ ^31^ ^]^ Copyright 2022, AAAS. Reproduced with permission.^[^ ^32^ ^]^ Copyright 2023, AAAS. Reproduced with permission.^[^ ^33^ ^]^ Copyright 2024, Springer Nature.

Field‐effect transistors (FETs) serve as the core building blocks for circuits responsible for signal processing and computation. Therefore, developing highly stretchable FETs is essential for realizing E‐skin (**Figure** **2**).^[^ ^21^, ^22^, ^23^ ^]^ To achieve intrinsic stretchability in these devices, the different components of the transistors—including the substrate, conductors (gate, source, drain), dielectrics, and semiconductors—must endure mechanical deformation while maintaining their electrical properties.^[^ ^24^, ^25^ ^]^ Among these, semiconductors are particularly important (Figure 1b), acting as an active channel through which current flows between the source and drain. FET performance is strongly dependent on the semiconductor properties, which directly affect the switching speed and current driving capability. However, although semiconductors play an important role in FETs, their low elasticity compared to other components limits the stretchability of the entire device. Moreover, low‐temperature processing (≈150 °C) is crucial for ensuring compatibility with stretchable polymer substrates. Therefore, understanding the properties and behavior of intrinsically stretchable semiconductor materials and developing suitable processing techniques is essential for advancing the field of stretchable electronics.

While several review papers offer in‐depth insights into the overall stretchable electronics landscape, comprehensive reviews that focus on the design and integration of semiconducting nanomaterials in intrinsically stretchable electronics are rarely reported. In this review, we focus on the technological trends in semiconducting nanomaterials for intrinsically stretchable FETs. We begin with an overview of stretchable FETs, discussing device architectures, material choices, and performance metrics. Next, the various semiconductor materials are categorized based on their nanostructures, providing detailed insights into the latest research progress, electrical properties, and mechanical characteristics. We then examine fabrication techniques and processes crucial for constructing intrinsically stretchable devices, followed by an exploration of recent advancements in the application of stretchable transistors. Finally, the critical challenges hindering the development of intrinsically stretchable electronic devices and potential solutions to overcome them are summarized, offering a forward‐looking perspective on future research directions.

## 2 Overview of Stretchable FETs

**Figure 3:**
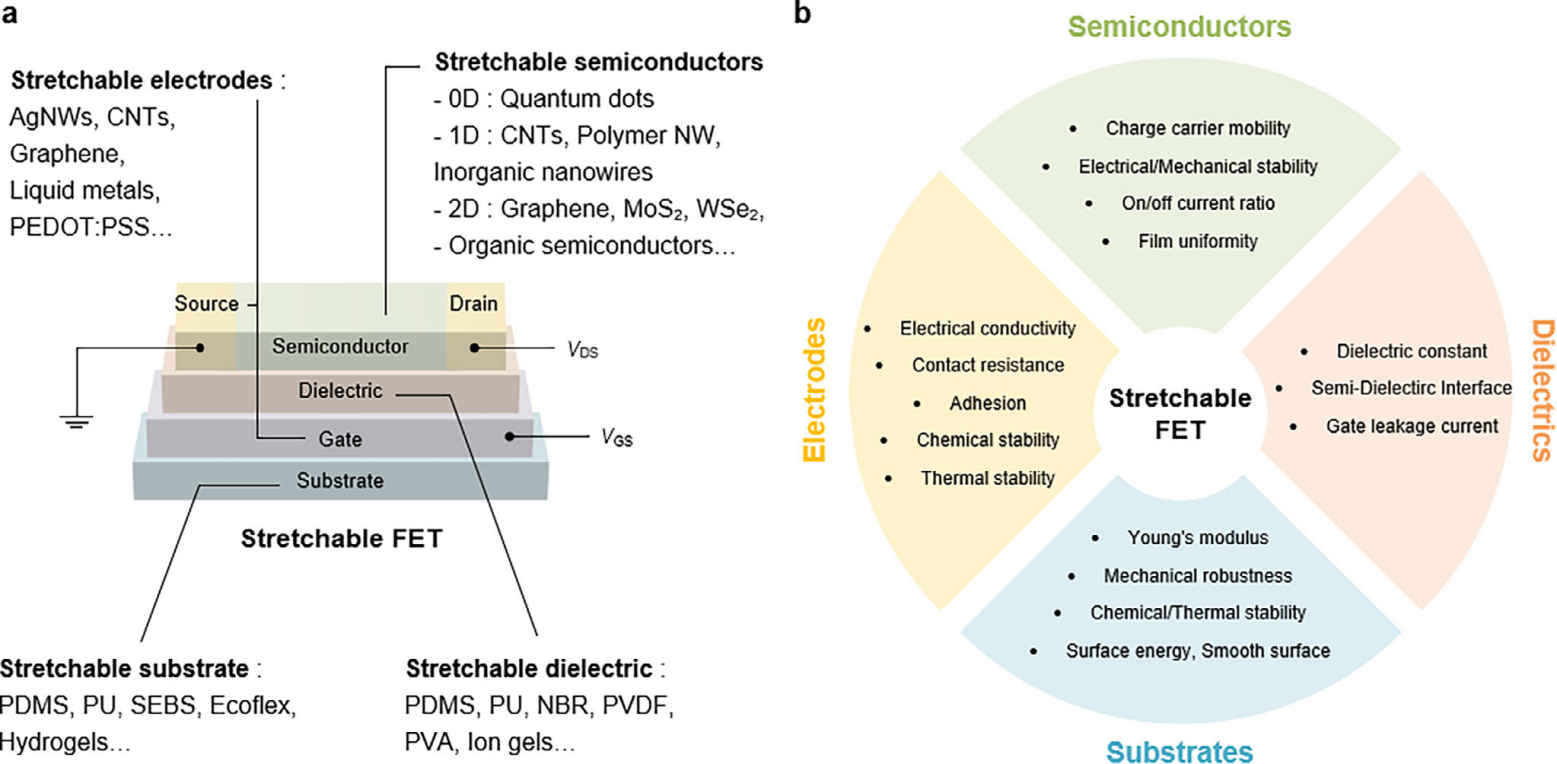
a) Schematic of stretchable FETs and representative materials for each component. b) Performance metrics of each component in stretchable FETs.

Stretchable FETs are fundamental device units for stretchable electronic circuits, controlling the flow of charge carriers through an electric field applied via a gate electrode. A typical FET consists of a substrate, electrodes, gate dielectric, and semiconductor layers, and operates by modulating the conductivity of the semiconductor channel located between the source and drain electrodes (**Figure** **3**). Applying a gate voltage induces charge accumulation or depletion at the semiconductor‐dielectric interface, switching the device on and off. In practical applications of stretchable electronics, FETs must withstand significant mechanical deformations without degrading electrical performance. Therefore, all layers must be able to maintain their electrical properties under mechanical deformation, requiring an optimal balance between mechanical resilience and stable device operation. In this section, we briefly introduce the materials and design strategies essential for each layer of stretchable FETs.

### 2.1 Stretchable Substrates

Stretchable substrates form the foundation layer supporting the electrodes, dielectrics, and semiconductor layers. Thermoplastic elastomers, such as polydimethylsiloxane (PDMS), polyurethane (PU), and styrene‐ethylene‐butylene‐styrene (SEBS), are commonly used as stretchable substrates owing to their low Young's modulus (<1 MPa).^[^ ^34^, ^35^, ^36^ ^]^ These materials can sustain strains exceeding 200% without mechanical failure and long‐term durability under stretched conditions. However, they exhibit a relatively low glass transition temperature (T_g_), thus limiting the temperature of the follow‐up process.^[^ ^37^, ^38^, ^39^, ^40^, ^41^ ^]^ Furthermore, deposition and doping processes should be selected considering the chemical stability of the substrate. To ensure manufacturing yield and uniformity, forming stretchable substrates with a smooth surface is crucial.^[^ ^42^, ^43^, ^44^, ^45^ ^]^

### 2.2 Stretchable Electrodes

The electrodes configuring the source, drain, and gate terminals in FETs are required for ensuring efficient charge injection and extraction. For stretchable applications, electrodes must exhibit both low electrical resistance and mechanical integrity, even under repeated deformation, bending, and torsion. A prevalent design strategy involves embedding conductive nanomaterials, such as silver nanowires (Ag NWs), carbon nanotubes (CNTs), graphene, and gold nanoparticles, within an elastomeric polymer matrix or depositing conductive films on stretchable substrates.^[^ ^46^, ^47^, ^48^, ^49^, ^50^ ^]^ Such materials enable the formation of percolating networks that preserve conductivity under strain. Additionally, conductive polymers like poly(3,4‐ethylenedioxythiophene):poly(styrene sulfonate) (PEDOT:PSS) are widely used owing to their solution‐processability and compatibility with stretchable substrates.^[^ ^51^ ^]^ More recently, emerging materials such as liquid metals (e.g., eutectic gallium‐indium (EGaIn) and Galinstan) have garnered attention, owing to their intrinsic stretchability, high conductivity, and minimal piezoresistivity.^[^ ^52^, ^53^, ^54^ ^]^

### 2.3 Stretchable Dielectrics

Stretchable dielectric layers are typically made from elastomeric polymers, similar to those used as substrates. Important electrical properties include a high dielectric constant and gate leakage current density; however, typical elastic dielectric polymers (such as PDMS and SEBS) exhibit low electrical dielectric properties. To enhance these properties, novel polymers have been synthesized using high‐κ monomers,^[^ ^55^, ^56^, ^57^ ^]^ and hybrid dielectric matrices have been explored, incorporating high‐dielectric nanoparticles into polymer matrices or using conductive metal particles.^[^ ^58^, ^59^ ^]^ Furthermore, electrolyte dielectrics, such as ionic liquids, ion‐gels, and polyelectrolytes, are a promising alternative, offering high areal capacitances (≈10 µF cm^−2^) by forming an electrical double layer under applied bias.^[^ ^60^, ^61^ ^]^

### 2.4 Stretchable Semiconductors

**Figure 4:**
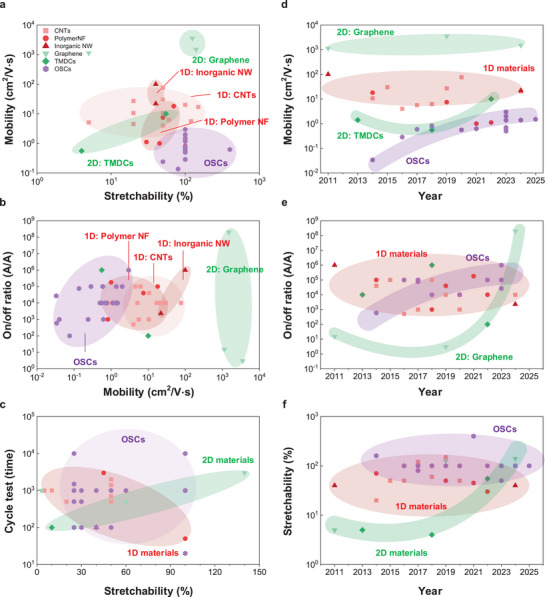
Comparison of intrinsically stretchable field‐effect transistor performance of semiconducting nanomaterials. a) Mobility as a function of stretchability. b) On/off ratio as a function of mobility. c) Cycle test time as a function of stretchability. d) Mobility, e) On/off ratio, and f) maximum stretchability as a function of year. Detailed values and their references are provided in Table 1.

Among the essential components of stretchable FETs, the semiconducting layer is arguably the most critical, as it forms the active channel where charge carriers are modulated by the gate electric field. Consequently, the intrinsic electronic properties of the semiconductor govern key device parameters, including carrier mobility, threshold voltage, on/off current ratio, and subthreshold swing. Despite recent progress, the development of intrinsically stretchable semiconductors remains fundamentally constrained by an inherent trade‐off between electrical performance and mechanical compliance. Achieving high charge carrier mobility typically requires a high degree of molecular ordering, strong intermolecular *π–π* stacking, or crystalline domains—all of which confer mechanical brittleness and poor strain tolerance.^[^ ^62^, ^63^ ^]^ In contrast, materials designed for mechanical deformability often exhibit low crystallinity and a soft, disordered microstructure, which inherently constrain charge transport efficiency.^[^ ^64^, ^65^ ^]^ This paradoxical relationship presents a significant design challenge in the pursuit of high‐performance stretchable semiconductor materials. Representative semiconducting materials and their electrical and mechanical properties are presented in **Figure** **4** and **Table** **1**.

**Table 1:** Summary of the performance parameters of representative semiconducting materials of intrinsically stretchable FETs.

Semiconducting materials (Dimension / Active layers)			Application	Substrate/Dielectric/S,D Electrode/Gate	Mobility^a)^ [cm^2^/V s]	On/off ratio [A/A]	Stretchability [%]	Strain cycle (strain [%])	Year	Reference
0D	CdSe/ZnS+PDPP2T		Phototransistor	SEBS/SEBS//Au/Au	−	−	30	1000 (30)	2022	[74]
	CPB+CONPHINE		Phototransistor	SEBS/SEBS/CNT/CNT	−	−	100	1000 (25)	2022	[75]
	CdSe/ZnS+CNT		Biomimetics	PDMS/PDMS+Ion gel/CNT/CNT	7.27^b)^ (h)	−	80	−	2023	[76]
	CPB+CONPHINE		Biomimetics	PDMS/PDMS+CQD/CNT/CNT	‐	−	100	1000 (50)	2024	[77]
	CdSe+CONPHINE		Phototransistor	SEBS/SEBS/Ag/Ag	−	−	50	10000 (50)	2024	[78]
1D	CNT		FET	PDMS/Ion gel/Cr,Au/Cr,Au	10.6^b)^ (h)	4.0 × 10^4^	20	1000 (10)	2014	[79]
			Display	PUA/PU‐PEG/AgNW‐PUA/AgNW‐PUA	30 (h)	10^4^	50	500 (20)	2015	[80]
			Digital computing	PI+PDMS/Al_2_O_3_/Au/Au	4.51 (h)	10^5^	20	‐	2015	[81]
			Digital computing	PDMS/BTO, PDMS/CNT/CNT	4 (h)	5.0 × 10^2^	50	1400 (50)	2016	[82]
			Display	SEBS/SEBS/Gr/Gr	5.6 (h)	10^3^	120	700 (50)	2017	[83]
			Sensory	SEBS/SEBS/CNT/CNT	6.18 (h)	10^5^	60	−	2018	[84]
			FET	PDMS/Ion gel/Au/Au	5.11^b)^ (h)	10^4^	5	1000 (5)	2019	[70]
			Digital computing	VHB/Ion gel/Au/Au	17^b)^ (h)	10^4^	150	−	2019	[85]
			Biomimetics	SEBS/Ion gel/PEDOT:PSS/CNT	27^b)^ (h)	10^4^	20	−	2019	[86]
			FET	PDMS/SEBS/CNT/CNT	10.45 (h)	10^3^	50	500 (50)	2020	[87]
			FET	PDMS/PUU/CNT/CNT	76.8 (h)	10^4^	50	2000 (50)	2020	[88]
			Sensory	SEBS/NBR+SEBS/CNT,Pd/PEDOT:PSS	20 (h)	10^4^	100	1000	2024	[33]
	Polymer nanofibers	P3HT	FET	SBS/Ion gel/Au/Au	18^b)^ (h)	10^5^	70	1500	2014	[89]
		FT4‐DPP	FET	SEBS/SEBS/CNT/EGain	0.82 (h)	10^3^	100	50 (100)	2018	[90]
		P3HT+CNT	Digital computing	PDMS/Ion gel/AuNP‐AgNW/AuNP‐AgNW	7.45^b)^ (h)	4.1 × 10^4^	50	100	2019	[91]
		DPPT‐TT	TFT array	PDMS/SEBS/Au,AgNF/AgNF	1 (h)	1.8 × 10^5^	45	3000 (45)	2021	[92]
		P3HT	Sensory	APTES‐PDMS/Ion gel/PEDOT:PSS/PEDOT:PSS	1.11^b)^ (h)	10^4^	30	−	2022	[93]
	Inorganic nanowires	SnO_2_	TFT array	PDMS, PI/PI/Au/Au	100 (h)	10^6^	40	−	2011	[94]
		Te	TFT array	SEBS/Ion gel/CNT/CNT	22^b)^ (h)	2.3 × 10^3^	40	100 (40)	2024	[72]
2D	Graphene		FET	PDMS/Ion gel/Gr/PEDOT:PSS	1131^b)^ (e)	15	5	1000 (3)	2011	[26]
			FET	Mylar/Ion gel/Cr,Au/Cr,Au	3544^b)^ (e)	3	125	500 (60)	2019	[95]
			FET	PDMS/PI/Gr/Gr	1500 (e)	2.0 × 10^8^	140	3000 (140)	2024	[96]
	TMDCs	MoS_2_	FET	PDMS/Ion gel/Au/Au foil	1.4^b)^ (e)	10^4^	5	−	2013	[97]
		MoS_2_	FET	PI+SU8/Al_2_O_3_/Gr/Cr,Pd	0.56 (e)	10^6^	4	−	2018	[98]
		MoS_2_	FET	‐/‐/Au/Au	10^b)^ (e)	10^2^	55	100 (10)	2022	[31]
	OSC	P3HT	FET	PUA/PU/CNT/EGain	0.034 (h)	5.9 × 10^2^	160	100 (40)	2014	[99]
		DPP+PDCA	TFT array	PDMS/PDMS/PEDOT:PSS+CNT/CNT	0.286 (h)	10^5^	100	500 (30)	2016	[100]
		PSe‐DPP	FET	SEBS/HFP‐PVP/AgNW/PEDOT:PSS	0.136 (h)	7.6 × 10^4^	80	1000 (40)	2017	[55]
		CONPHINE	Display	SEBS/SEBS/CNT/CNT	0.59 (h)	10^5^	100	1000 (25)	2017	[27]
		CONPHINE	Digital computing	SEBS/SEBS/CNT/CNT	0.78 (h)	10^4^	100	1000 (100)	2018	[28]
		DPP‐TVT	TFT array	SEBS/PDMS/Au/Au	0.076 (h)	10^2^	100	100 (50)	2019	[29]
		PII2T	Display	PDMS/PFPE‐DMA/PEDOT:PSS+CNT/PEDOT:PSS+CNT	0.56 (h)	10^4^	100	1000 (30)	2020	[101]
		CONPHINE	Digital computing	SEBS/SEBS/CNT/CNT	0.62 (h)	10^3^	400	1000 (100)	2021	[30]
		CONPHINE	TFT array	SEBS/SEBS/Ag/Ag	0.288 (h)	10^5^	100	10000 (100)	2022	[102]
		CONPHINE	TFT array	PAAm+SEBS/SEBS/CNT/CNT	0.7 (h)	10^4^	100	1000 (100)	2023	[103]
		IDT‐BT	FET	PDMS/PDMS/PEDOT:PSS+CNT/PEDOT:PSS+CNT	2.98 (h)	10^6^	100	1500 (25)	2023	[104]
		N2200	TFT array	PDMS/PU/AgNW/EGain	0.24 (e)	10^3^	50	1000 (50)	2023	[105]
		DPPTT+PFDT	FET	PDMS/SEBS/CNT/CNT	1.38 (h)	10^5^	100	1000 (50)	2023	[106]
		N2200/SEBS	FET	PDMS/SEBS/PEDOT:PSS+CNT/PEDOT:PSS+CNT	0.033 (h)	2.7 × 10^4^	50	100 (25)	2023	[107]
		CONPHINE	Sensory	SEBS/SEBS/Ag/Ag	0.5 (h)	10^4^	100	−	2023	[108]
		CONPHINE	Display	SEBS/SEBS+CQD/CNT/CNT	0.6 (h)	10^5^	100	1000 (25)	2023	[56]
		CONPHINE	Biomimetics	PDMS/NBR+SEBS/CNT/CNT	2 (h)	10^5^	100	1000 (60)	2023	[32]
		IDT‐BT	TFT array	PDMS/PUU/PEDOT:PSS+CNT/PEDOT:PSS+CNT	1.39 (h)	10^4^	100	10000 (25)	2024	[57]
		PPT	FET	PDMS/PDMS/CNT/CNT	1.17 (h)	10^4^	100	500 (25)	2024	[109]
		DPP‐DTT+SHP	TFT array	SEBS/PDMS‐MPU/CNT/CNT	0.04 (h)	10^3^	30	100 (30)	2025	[110]
		TDPP‐Se	Sensory	SEBS/NBR+SEBS+NBR‐C/CNT/CNT	1.5 (h)	10^4^	100	20 (100)	2025	[111]

^a)^ h): hole field effect mobility, e): electron field effect mobility;

^b)^ charge carrier mobility extracted using ion gel for dielectric.

Organic semiconductors (OSCs) represent the most extensively studied class of stretchable semiconductor materials.^[^ ^64^ ^]^ Originally developed within the organic field‐effect transistors (OFETs) community for high‐performance applications,^[^ ^66^, ^67^, ^68^ ^]^ organic semiconductors have been actively explored for stretchable electronics, owing to their inherent mechanical softness, compatibility with solution‐based low‐temperature processing, and excellent integration with stretchable dielectric layers,^[^ ^20^, ^69^ ^]^ which afford significant improvements in mechanical stretchability, device manufacturing yield, and integration density. As shown in Figure 4a, while OSCs inherently offer excellent stretchability (≈50–300%), they exhibit relatively lower field‐effect mobility (≈10^−2^–1 cm^2^ V s^−1^) compared with other semiconductor materials, thus limiting their potential for achieving high‐speed and high‐current operation.

1D semiconducting materials, including CNTs and nanowires, have emerged as promising candidates owing to their superior carrier mobility and suitability for solution processing. Alignment strategies for 1D materials have significantly improved the uniformity of the network and reduced the inter‐junction resistance, further enhancing electrical performance.^[^ ^70^, ^71^, ^72^ ^]^ Semiconducting 1D network films exhibit good stretchability (≈100%). In terms of electrical properties, polymer nanofibers have better mobility (≈0.1–10 cm^2^ V s^−1^) than OSCs, while CNTs and inorganic nanowires exhibit better charge transport properties (≈10–100 cm^2^ V s^−1^). Recent studies on the use of CNTs have reported high electrical field effect mobility (≈20 cm^2^ V s^−1^) and high device density (>100,000 transistors per cm^2^).^[^ ^33^ ^]^ However, achieving isotropic mechanical stretchability remains challenging, as the anisotropic nature of the aligned CNT networks often leads to strain localization and electrical degradation under multidirectional stretching.

2D semiconducting materials, including graphene and transition metal dichalcogenides (TMDs), offer additional pathways toward high‐performance stretchable electronics. Their atomic‐scale thickness confers outstanding mechanical flexibility, while their band structures enable high electron mobility.^[^ ^73^ ^]^ However, graphene intrinsically lacks a bandgap, resulting in poor on/off current ratios and necessitating careful doping or bandgap engineering for FET applications. As shown in Figure 4a,b, graphene shows extremely high mobility (>1,000 cm^2^ V s^−1^) and excellent stretchability (≈100%), but relatively poor on/off ratio (≈10^1^), with few exceptions. In contrast, TMDs such as MoS_2_ exhibit sizable band gaps and high carrier mobility; however, studies on stretchable devices remain limited due to the challenges associated with the high‐temperature growth process and the difficulty in achieving large‐area uniformity.

While oxide semiconductors and metal halide perovskites have emerged as high‐performance materials in conventional electronics, their intrinsic brittleness and limited mechanical deformability pose fundamental challenges for integration into intrinsically stretchable electronics. Oxide semiconductors such as indium gallium zinc oxide (IGZO) and zinc oxide (ZnO) offer high carrier mobility and operational stability, yet their rigid crystal structures are inherently incompatible with repeated mechanical strain. Similarly, although hybrid organic–inorganic perovskites exhibit favorable optoelectronic properties and processability, they are prone to ion migration, environmental degradation, and fracture under strain. To address these limitations, materials strategies such as nanocomposite embedding, polymer blending, and multilayer engineering have been developed to enhance mechanical compliance. More recently, viscoelastic perovskites, stretchable heterojunctions, and 2D nanosheet‐based architecture have shown promise in bridging the mechanical mismatch. While these approaches do not fully overcome the intrinsic mechanical limitations of oxides and perovskites, they offer a pathway toward functionally stretchable devices. From a materials standpoint, future efforts must focus on discovering new compositions with inherently deformable lattice structures, suppressing ion migration, and improving long‐term stability under mechanical stress to unlock the full potential of these material systems in next‐generation stretchable electronics.

Stability of stretchable test is another critical factor for stretchable electronics. However, stretchable performance is not solely determined by the semiconductor material itself; it also significantly depends on various structural parameters and the mechanical properties of additional device components, including substrates, dielectrics, and electrodes. Figure 4c summarizes and compares reported stretchability and cyclic stability of diverse semiconducting nanomaterials. On average, stability evaluations were performed at ≈50% strain over ≈1,000 cycles. Notably, OSCs and CNTs exhibit a relatively wide distribution in cycle performance, reflecting their inherent mechanical adaptability and variability depending on structural designs or composite formulations.

The trends of key performance metrics of stretchable transistors are presented in Figure 4d–f, highlighting the progress in device performance across different years. The charge carrier mobility shows a gradual improvement for OSC, but challenges to surpass the intrinsic mobility limits of OSC. The on/off ratio maintains relatively consistent values ≈10^4^–10^6^, there is still room for further optimization. Stretchability has now reached a stable level of ≈100%. Therefore, rather than pursuing further enhancement of stretchability, future research should focus on resolving the trade‐off between mechanical and electrical performance to achieve balanced and reliable device operation.

Consequently, developing new stretchable semiconductor materials requires both high electrical performance and compatibility with low‐temperature and suitable fabrication processes. Furthermore, innovative approaches that transcend the limitations of existing materials through novel material design, nanostructuring, and advanced process engineering are essential to fully realize the next generation of intrinsically stretchable FETs. In Section 3, recent advancements in nanostructured semiconducting materials are discussed in detail, classified according to their dimensionality (0D, 1D, 2D, and OSCs), particularly focusing on their electrical and mechanical properties.

## 3 Semiconducting Nanomaterials for Stretchable FETs

### 3.1 0D Semiconducting Nanomaterials

0D nanomaterials, with all dimensions confined to the nanometer scale (typically less than 100 nm), exhibit unique properties arising from quantum confinement effects. These effects significantly alter their electronic and optical properties compared to bulk materials. Their high surface area to volume ratio enhances their chemical reactivity and interaction with other materials, while their quantized electronic states lead to discrete energy levels. Semiconducting 0D materials include quantum dots (QDs, e.g., CdSe, PbS, and perovskite QDs), nanoparticles (e.g., TiO_2_ and ZnO), and colloidal nanocrystals (e.g., silicon and CIS nanocrystals), each exhibiting unique size‐dependent optical and electronic properties.^[^ ^112^ ^]^

**Figure 5:**
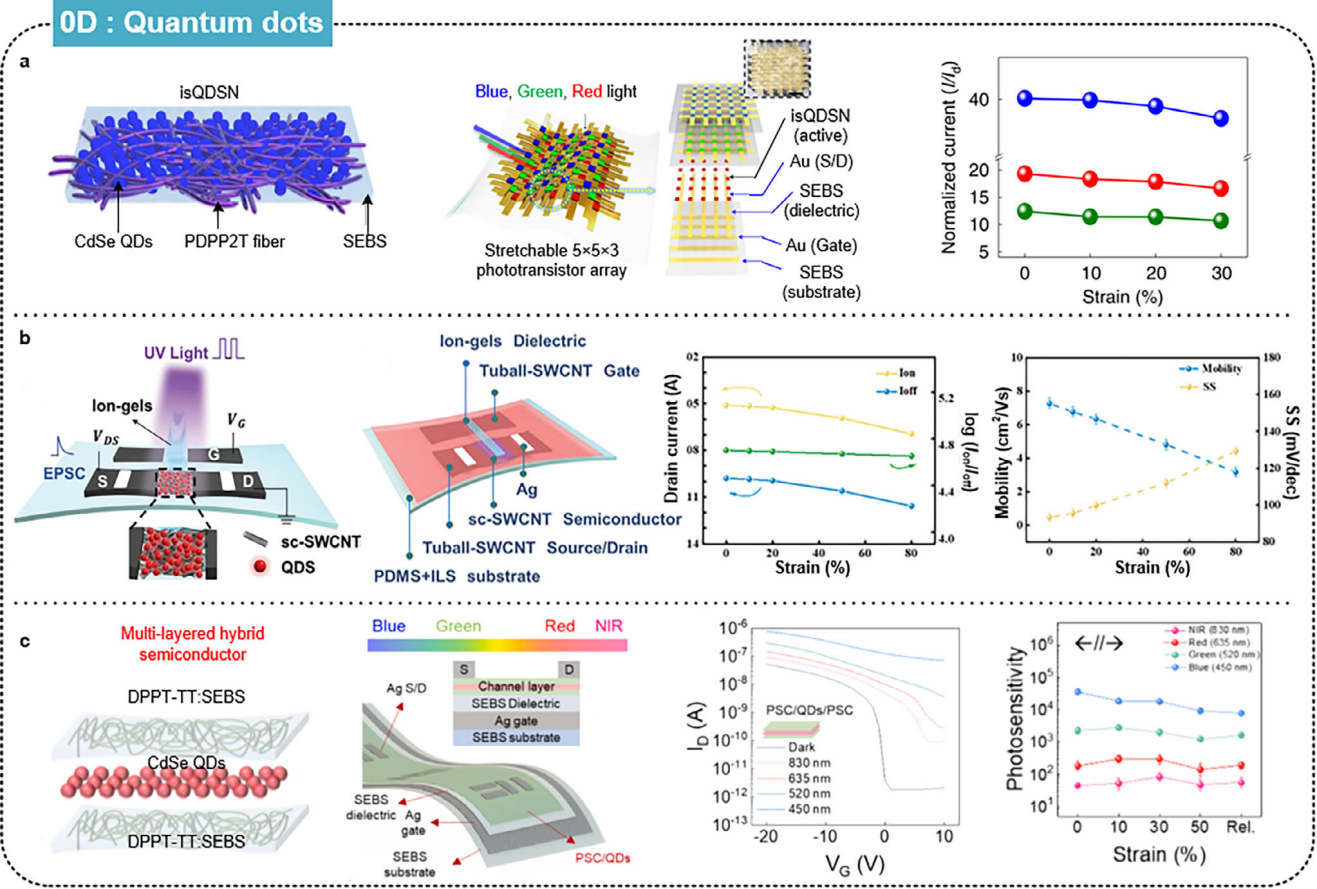
Intrinsically stretchable FETs based on 0D semiconducting materials. a) Multiplexed isQDSN‐based stretchable phototransistor arrays. Reproduced with permission.^[^ ^74^ ^]^ Copyright 2022, Springer Nature. b) Stretchable optoelectronic synaptic transistor arrays using CNTs with CdSe/ZnS quantum dots. Reproduced with permission.^[^ ^76^ ^]^ Copyright 2023, Wiley‐VCH. c) Intrinsically stretchable phototransistors with polymer‐QD‐polymer multi‐layered hybrid films. Reproduced with permission.^[^ ^78^ ^]^ Copyright 2024, Elsevier.

QDs are typically employed as active layers in stretchable optoelectronic devices. Rather than being used alone, QDs are typically blended with other semiconducting materials to form active layer films. Song et al. reported intrinsically stretchable QD‐based semiconducting nanocomposites (isQDSN) for shape‐tunable multiplexed phototransistor arrays (**Figure** **5**a).^[^ ^74^ ^]^ Size‐tunable CdSe/ZnS core/shell QDs enabled multispectral photo absorption, with the semiconducting polymer PDPP2T‐TT‐OD facilitating charge transport within the elastomeric SEBS matrix. This combination provides high photosensitivity and ensures the material can endure significant mechanical strain. The 5 × 5 × 3 multiplexed phototransistor array, capable of detecting red, green, and blue light, maintained a stable performance under 30% strain and showed minimal changes in photo response after 1,000 stretching cycles.

To address the issue of high power consumption in transitional stretchable devices, Xie et al. fabricated a SWCNT‐QDs mixture for stretchable optoelectronic synaptic transistor arrays (Figure 5b).^[^ ^76^ ^]^ These CdSe/ZnS devices, integrated with ion‐gel‐based transistors, exhibited high on/off ratios (up to 10^5^), negligible hysteresis, and low subthreshold swing. After 20% vertical and horizontal strain stretching, they maintained their electronic performance with small degradations (12.4% and 6.4% in carrier mobility) and exhibited ultra‐low power consumption (15.38 aJ) as a synaptic characteristic under light stimulation.

To enhance the durability of the QD optoelectronic properties under strain, Nam et al. proposed multi‐layered hybrid films with a polymer‐QD‐polymer structure for intrinsically stretchable phototransistors (Figure 5c).^[^ ^78^ ^]^ The top and bottom layers comprised DPPT‐TT and SEBS, while the middle layer consisted of CdSe/ZnS QDs. These hybrid films exhibited high photosensitivity and photoresponsivity across red, green, blue, and NIR light, maintaining their performance under strains up to 50% and after 10,000 stretching cycles. Furthermore, their multi‐layered structure enhanced the mechanical robustness and reliable device performance.

### 3.2 1D Semiconducting Nanomaterials

#### 3.2.1 Carbon Nanotubes

**Figure 6:**
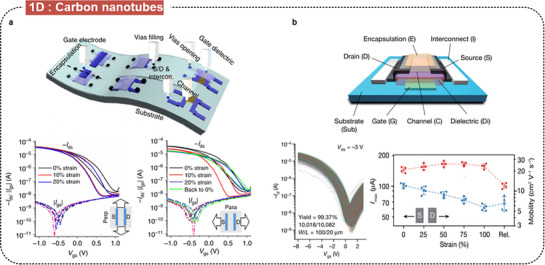
Intrinsically stretchable FETs based on semiconducting carbon nanotubes. a) Inkjet‐printed stretchable CNT synaptic transistor arrays. Reproduced with permission.^[^ ^86^ ^]^ Copyright 2019, Springer Nature. b) High‐speed and large‐scale CNT stretchable integrated circuits. Reproduced with permission.^[^ ^33^ ^]^ Copyright 2024, Springer Nature.

CNTs are the most representative 1D semiconducting materials, owing to their superior tensile strength, elastic modulus, electrical conductivity, thermal conductivity, and chemical stability. Depending on their chirality and diameter, CNTs exhibit either metallic or semiconducting behavior, being versatile for use in both electrodes and active layers in electronic devices. Molina‐Lopez et al. fabricated inkjet‐printed CNT stretchable synaptic transistor arrays (**Figure** **6**a).^[^ ^86^ ^]^ The transistors were fabricated via step‐by‐step inkjet‐printing using PEDOT:PSS ink as electrodes, MWCNT inks for filling and interconnections, fluorinated polymer PVDF‐HFP inks as the dielectric, and sc‐SWCNT inks as the channel layers. This non‐contact and maskless printing method offers efficient patterning of chemically sensitive materials over large areas. Furthermore, the density of the sc‐SWCNT networks can be precisely controlled by adjusting the number of printing passes, enabling tunable surface coverage and optimized electronic properties. The developed transistors showed low‐voltage operation, high mechanical flexibility, and robust electrical performance, with mobilities up to 30 cm^2^ V s^−1^ and stable operation under strains up to 20%.

Intrinsically stretchable integrated circuits are reported to exhibit low electrical characteristics (a charge‐carrier mobility of ≈1 cm^2^ V s^−1^) and integration scale (for example, 54 transistors per circuit). Recently, Zhong et al. reported high‐speed and dense large‐scale intrinsically stretchable integrated circuits with high‐purity semiconducting CNTs (Figure 6b).^[^ ^33^ ^]^ They achieved high performance and scalability by integrating S‐CNTs with low‐contact‐resistance source‐drain electrodes made from metallic CNT/palladium (M‐CNT/Pd) and high‐κ elastic polymer dielectric. These transistors exhibited a field‐effect mobility of over 20 cm^2^ V s^−1^ under 100% strain and maintained a high device yield of more than 99.3%. With a device density of 100,000 transistors per cm^2^, the developed transistors demonstrated high drive currents and an operation speed of ring oscillator exceeds 1 MHz.

#### 3.2.2 Polymer Nanofibers

Polymer nanofibers based on organic materials are characterized by their high mechanical strength, flexibility, porosity, and potential for functionalization. Transforming from a 3D polymer nanostructure to a 1D fiber structure can help enhance the electrical properties, while the inherent biocompatibility, biodegradability, chemical, and thermal stability of nanofibers provide significant advantages in the development of stretchable transistors.

**Figure 7:**
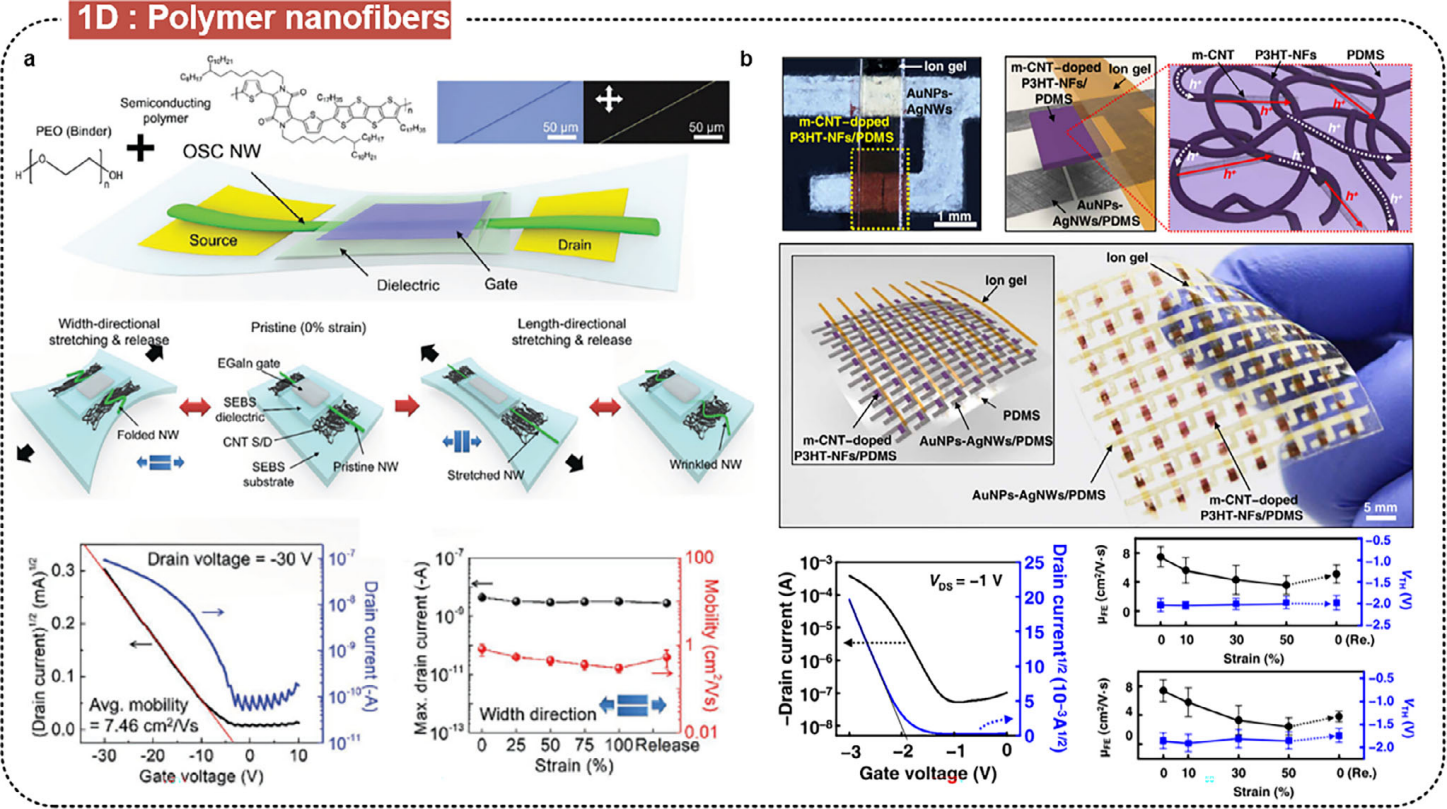
Intrinsically stretchable FETs based on semiconducting polymer nanofibers. a) Deformable organic nanowires FETs. Reproduced with permission.^[^ ^90^ ^]^ Copyright 2018, Wiley‐VCH. b) Intrinsically stretchable semiconductor of the m‐CNT‐doped P3HT‐NFs/PDMS‐based rubbery transistors. Reproduced with permission.^[^ ^91^ ^]^ Copyright 2019, AAAS.

The synthetic method of polymer nanofibers plays an important role in the electronic and mechanical properties of the fabricated transistors. Lee et al. synthesized deformable polymer nanofibers using the electrospinning method (**Figure** **7**a).^[^ ^90^ ^]^ The nanofibers comprised a blend of the semiconducting polymer FT4‐DPP and biocompatible polymer PEO in a 7:3 ratio, with an average diameter of 675 nm. During electrospinning synthesis, these nanofibers are aligned, which could enhance charge transfer compared to conventional polymer systems. The aligned nanofiber FETs showed high field‐effect mobilities of up to 8.45 cm^2^ V s^−1^ on a SiO_2_ rigid substrate with a high‐κ polymer dielectric. They maintained electrical performance under significant mechanical strain, with robust operation under 100% tensile and compressive strain, and average mobilities ≈0.78 cm^2^ V s^−1^ on a stretchable substrate. Recently, Zhang et al. proposed a new approach for synthesizing polymer nanofibers,^[^ ^113^ ^]^ the flow‐enhanced crystallization (FLEX) method. Compared with conventional wet‐spinning synthetic methods, FLEX can enhance the mechanical strength and electronic performance through decomposition, slow antisolvent diffusion, and post‐drawing processes. The fabricated fibers exhibit higher tensile strength (>200 MPa), toughness (>80 MJ m^−3^), and stretchability (>50%) than traditional processed polymer fibers.

The development of nanofiber‐based transistors has evolved from single‐device fabrication to the realization of integrated arrays and circuits, marking a significant step toward the scalable and complex architectures required for advanced electronic applications. Sim et al. reported a fully rubbery integrated transistor array using high‐mobility, intrinsically stretchable semiconductor materials (Figure 7b).^[^ ^91^ ^]^ The P3HT nanofibers doped with metallic CNTs were used in the channel layer embedded in a PDMS matrix. The embedded composite achieved a high carrier mobility (≈9.76 cm^2^ V s^−1^) owing to the enhanced charge transport efficiency facilitated by the metallic CNT doping, with stable electrical properties under 50% mechanical strain. Furthermore, Kim et al. fabricated stretchable transistor arrays based on metal (Au and Ag nanofibers) and DPP‐based semiconducting nanofibers via the electrohydrodynamic (EHD) printing method.^[^ ^92^ ^]^ The highly integrated nanofiber‐based FET array showed high optical transparency (>90%), device integrated density (10 FETs/mm^2^), uniformity (≈90%), mobility (1.0 cm^2^ V s^−1^), and mechanical deformability (>45%).

#### 3.2.3 Inorganic Nanowires

Inorganic nanowires are made from various materials such as metals (e.g., gold and silver), semiconductors (e.g., silicon, germanium, and gallium arsenide), and oxides (e.g., zinc oxide and titanium dioxide), possessing distinct properties that render them suitable for numerous applications. These nanowires exhibit high electrical conductivity, superior mechanical strength, and excellent thermal stability. For instance, owing to their high carrier mobility, excellent scalability, and established compatibility with existing semiconductor technologies, silicon‐based nanomaterials are widely used in developing stretchable FETs.^[^ ^114^, ^115^ ^]^ Zinc oxide nanowires, with their piezoelectric and photoconductive properties, are frequently employed in sensors and photovoltaic devices, thus enhancing sensitivity and efficiency. Gold and silver nanowires are utilized in transparent conductive films for electrodes, exhibiting exceptional electrical conductivity and flexibility.^[^ ^92^ ^]^ Furthermore, germanium nanowires have shown promise in lithium‐ion batteries, significantly improving their charge capacity and cycling stability. However, research in this area remains relatively limited because only a few semiconductor materials are technically viable for intrinsically stretchable transistors.

**Figure 8:**
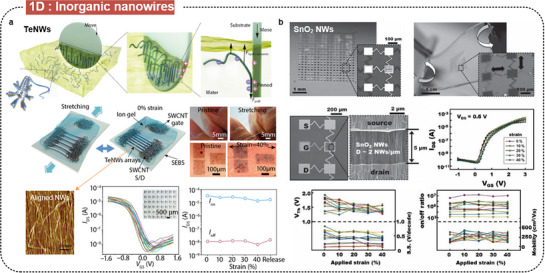
Intrinsically stretchable FETs based on inorganic semiconducting nanowires. a) Stretchable TFTs with wafer‐scale alignment of TeNWs. Reproduced with permission.^[^ ^72^ ^]^ Copyright 2024, AAAS. b) Stretchable FET array of suspended SnO_2_ NWs. Reproduced with permission.^[^ ^94^ ^]^ Copyright 2011, Wiley‐VCH.

Among single‐component nanowires, Zhao et al. recently reported the synthesis of tellurium nanowires (TeNWs) using a scalable hydrothermal method, producing nanowires with diameters of 9 ± 0.8 nm and lengths of 103 ± 38 µm (**Figure** **8**a).^[^ ^72^ ^]^ The developed TeNWs were aligned on substrates using a biomimetic lock‐and‐shear strategy, mimicking the alignment of jellyfish tentacles, resulting in high‐density, well‐aligned arrays. The resulting stretchable TeNW TFTs demonstrated impressive performance, with mobilities over 22 cm^2^ V s^−1^ and on/off ratios over 10^3^ with ion gel dielectric. These TeNW‐TFTs maintained their performance under strains up to 40%. Furthermore, Shin et al. synthesized tin oxide (SnO_2_) nanowires using a CVD method, involving a gold film catalyst at 750 °C (Figure 8b).^[^ ^94^ ^]^ These nanowires were integrated into stretchable FETs that demonstrated high performance, with field‐effect mobilities of ≈100 cm^2^ V s^−1^, current on/off ratios of ≈10^6^, SS of ≈0.5 V dec^−1^, and stretchability up to 40%.

### 3.3 2D Semiconducting Nanomaterials

#### 3.3.1 Graphene

Graphene comprises a single layer of carbon atoms arranged in a 2D honeycomb lattice, exhibiting unique properties owing to its atomic‐scale thickness and planar structure. Furthermore, it possesses exceptional tensile strength, high elastic modulus, superior electrical conductivity, remarkable thermal conductivity, and excellent chemical stability. The high carrier mobility and conductivity of graphene make it highly attractive for a wide range of electronic applications, including transistors, sensors, and transparent conductive films. In stretchable electronic devices, the high conductivity and mechanical flexibility of graphene are leveraged to develop components such as thin‐film transistors, sensors, and transparent conductive films. These graphene‐based devices can maintain their performance under significant mechanical strain, making them promising candidates for wearable electronics, flexible displays, and bio‐integrated devices. However, a major limitation of semiconducting graphene is its zero bandgap, which results in poor on/off current ratios and restricts its effectiveness in applications requiring a clear distinction between on and off states. Achieving the desired electronic properties often requires doping or functionalization; however, uniformly controlling the type and level of doping across large areas can lead to inconsistencies in device performance. Additionally, integrating graphene with other materials in a TFT stack is challenging due to differences in thermal expansion, chemical compatibility, and mechanical properties, making it difficult to ensure strong adhesion and minimize interface defects.

**Figure 9:**
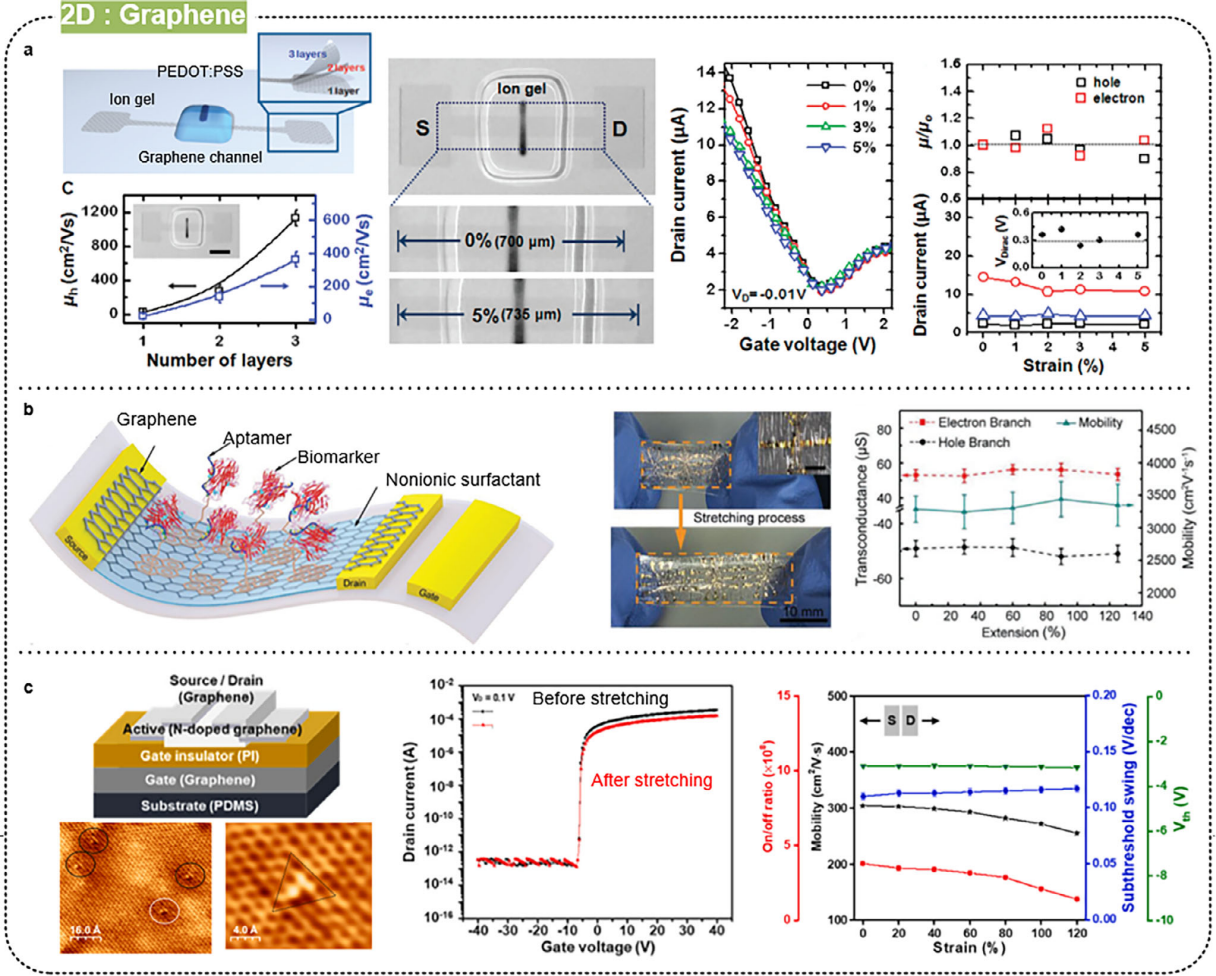
Intrinsically stretchable FETs based on graphene. a) Stretchable graphene transistors with printed ion gel and PEDOT:PSS electrodes. Reproduced with permission.^[^ ^26^ ^]^ Copyright 2011, American Chemical Society. b) Ultraflexible and stretchable graphene field‐effect transistor nanosensors. Reproduced with permission.^[^ ^95^ ^]^ Copyright 2019, Wiley‐VCH. c) Stretchable n‐doped graphene‐based FETs. Reproduced with permission.^[^ ^96^ ^]^ Copyright 2024, Elsevier.

Lee et al. developed stretchable graphene transistors using a combination of monolithically patterned graphene films and a low‐temperature printing process for dielectrics and gate electrodes (**Figure** **9**a).^[^ ^26^ ^]^ Graphene was synthesized on a copper foil using CVD and transferred onto rubber substrates. The ion gel dielectric and PEDOT:PSS electrodes were printed using an aerosol jet printing technique, enabling high‐resolution patterns and compatibility with various functional inks. These stretchable transistors exhibited high performance, with hole and electron mobilities of 1188 ± 136 and 422 ± 52 cm^2^ V s^−1^, respectively, maintaining stable operation under strains up to 5% even after 1000 cycles. Wang et al. developed a stretchable biosensor based on graphene FETs (Figure 9b).^[^ ^95^ ^]^ The graphene monolayer was functionalized with aptamer molecules specific to the target biomarker, enabling changes in carrier concentration upon binding with the biomarker. The nanosensor maintained consistent electrical properties and biomarker detection capabilities even under extreme deformations, such as rolling on surfaces with radii as low as 40 µm, twisting angles from −180° to 180°, and stretching up to 125%. Furthermore, it demonstrated high selectivity and a low limit of detection (LOD, 5 × 10^−12^ M) for TNF‐α, an inflammatory cytokine biomarker.

Developing doping methods to lower the off‐current in graphene transistors remains crucial. Jung et al. recently reported a scalable direct‐growth approach for fabricating n‐doped graphene‐based FET at low temperatures (Figure 9c).^[^ ^96^ ^]^ The n‐doped graphene FET exhibited enhanced stretchability up to 10% and cycle stability of ≈5,000 times under 7% tensile strain. When subjected to 120% parallel and 140% perpendicular stretching strains, the FET mobility showed a minimal decrease of ≈15% from its original value of ≈300 cm^2^ V s^−1^. The real *I*–*V* characteristics of FETs did not show any notable variations, except for a slight decrease in the on‐current after 140% perpendicular stretching strain. This approach offers a promising solution to the zero‐bandgap limitation of graphene for stretchable electronic applications.

#### 3.3.2 Transition Metal Chalcogenides (TMDCs)

2D Transition metal chalcogenides (TMDCs) are a class of materials with unique properties, being highly attractive for both fundamental studies and various applications, including nanoelectronics, nanophotonics, sensing, and actuation at the nanoscale.^[^ ^116^, ^117^, ^118^, ^119^ ^]^ These materials, denoted as MX_2_ (where M is a transition metal such as Mo or W and X is a chalcogen such as S, Se, or Te), have attracted attention for their potential in high‐performance devices. TMDCs possess atomic‐scale thickness, direct bandgap, strong spin‐orbit coupling, and excellent electronic and mechanical properties. Such features make them highly promising for fundamental research and applications in high‐end electronics, spintronics, optoelectronics, energy harvesting, flexible electronics, DNA sequencing, and personalized medicine.^[^ ^75^, ^120^, ^121^ ^]^

TMDCs are synthesized using various methods, including top‐down approaches like mechanical and liquid exfoliation^[^ ^122^, ^123^, ^124^, ^125^, ^126^, ^127^, ^128^, ^129^ ^]^ and bottom‐up techniques such as CVD,^[^ ^130^, ^131^ ^]^ metal–organic chemical vapor deposition (MOCVD), atomic layer deposition (ALD), and molecular beam epitaxy (MBE). TMDCs hold great promise owing to their flexibility, direct bandgap, and superior electronic properties, which are ideal for applications in flexible electronics and sensors.^[^ ^132^, ^133^, ^134^, ^135^ ^]^ However, several challenges remain, including maintaining high carrier mobility, low contact resistance, and mechanical stability under strain, as well as achieving scalable, high‐quality film production. Overcoming these challenges is crucial for the practical implementation of TMDC‐based stretchable electronics.^[^ ^136^, ^137^ ^]^

**Figure 10:**
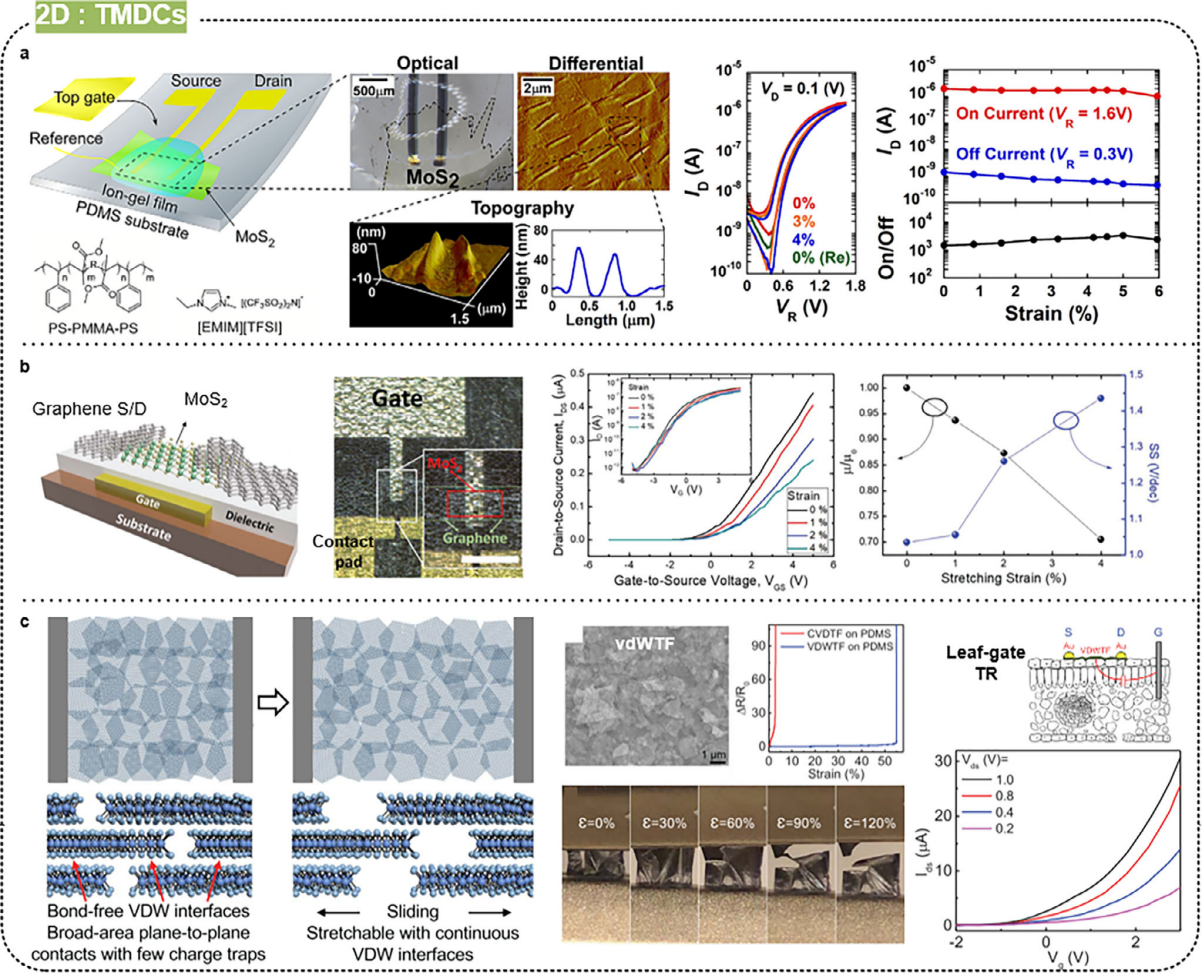
Intrinsically stretchable FETs based on TMDCs. a) Stretchable MoS_2_ TFTs using ion‐gel dielectric. Reproduced with permission.^[^ ^138^ ^]^ Copyright 2013, AIP Publishing LLC. b) Stretchable MoS_2_ TFTs with graphene electrodes. Reproduced with permission.^[^ ^98^ ^]^ Copyright 2018, The Royal Society of Chemistry. c) Highly stretchable van der Waals thin films. Reproduced with permission.^[^ ^31^ ^]^ Copyright 2022, AAAS.

Pu et al. reported the first stretchable MoS_2_ TFTs using elastic ion‐gel gate dielectrics (**Figure** **10**a).^[^ ^138^ ^]^ The MoS_2_ thin films were synthesized via CVD and transferred to the substrate. These transistors exhibited an electron mobility of 1.40 cm^2^ V s^−1^ and an on/off current ratio of 10^4^, maintaining stable performance under 5% strain. Park et al. harnessed the interface properties of 2D materials by integrating graphene electrodes on CVD‐grown MoS_2_ stretchable TFTs (Figure 10b).^[^ ^98^ ^]^ The MoS_2_ and graphene films were transferred using polystyrene‐assisted and thermal release‐assisted methods, respectively. These TFTs exhibited an electron mobility of 0.56 cm^2^ V s^−1^, an on/off current ratio of ≈10^6^, and stretchability of ≈4%.

Even though the elasticity was lower compared with the theoretical values, Yan et al. developed highly stretchable van der Waals thin films (VDWTF) composed of staggered 2D nanosheets with bond‐free VDW interfaces, synthesized via a solution process (Figure 10c).^[^ ^31^ ^]^ These VDW thin films demonstrated a remarkable stretchability of up to 60%, a significant improvement over the less than 5% stretchability of conventional CVD films.

### 3.4 Organic Semiconductors

#### 3.4.1 Backbone Engineering

In the field of stretchable polymer semiconductors, the molecular design and structure of the conjugated polymer backbones are crucial, directly affecting the mechanical properties and charge transport capabilities of the resulting materials. Conjugated polymer backbones are typically engineered using donor‐acceptor (D‐A) structures, where alternating donor and acceptor units create a backbone that facilitates efficient charge transport while maintaining flexibility. Various modifications of the backbone, focusing on reducing the sustained length and increasing the flexibility of the polymer chains, have been studied to improve elasticity.^[^ ^139^ ^]^ In this section, we will provide some examples of such studies based on the backbone design.

**Figure 11:**
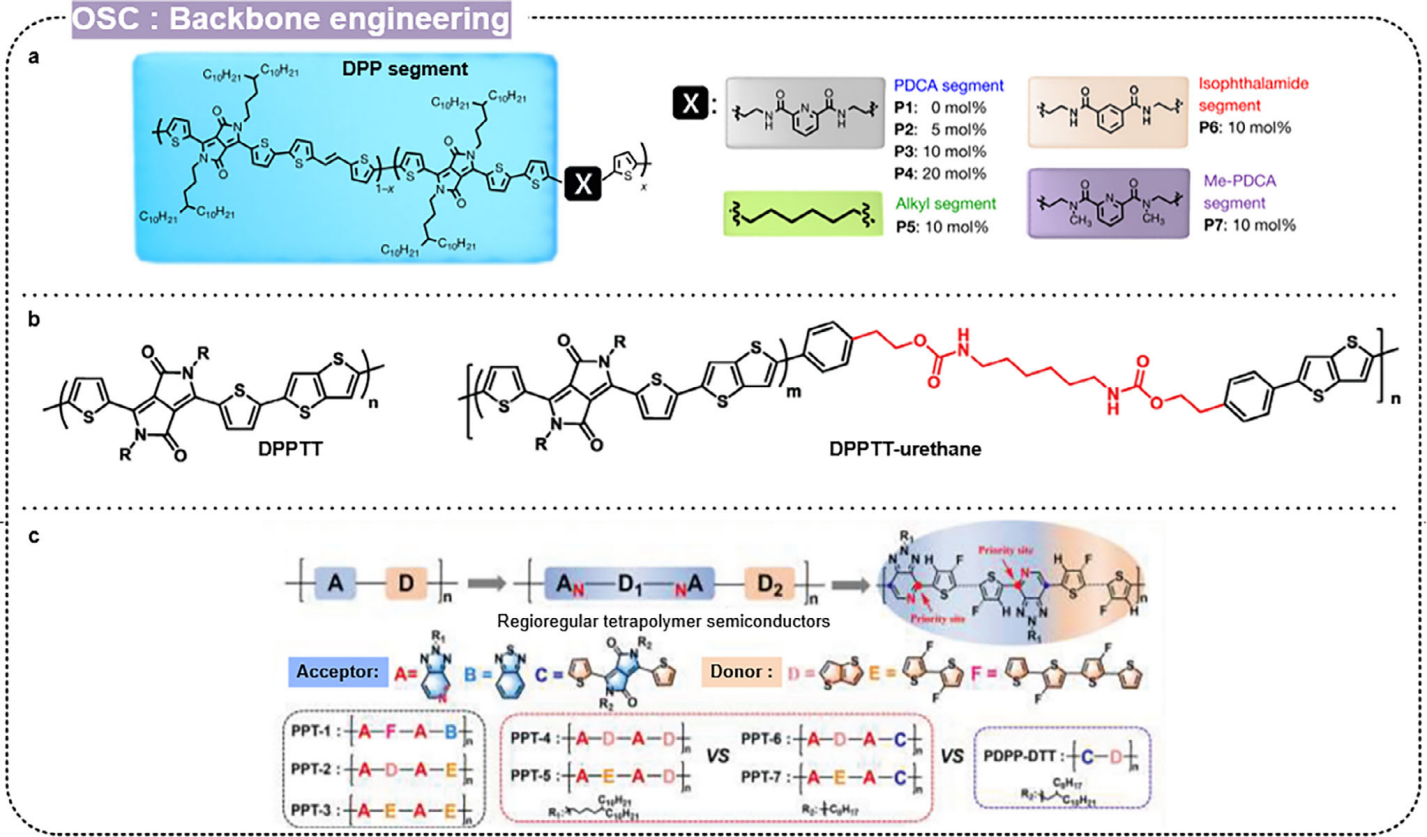
Molecular design of stretchable semiconducting polymers by backbone engineering. a) DPP‐based intrinsically stretchable and healable semiconducting polymer. Reproduced with permission.^[^ ^100^ ^]^ Copyright 2016, Springer Nature. b) Regional conjugation of aligo‐DPPTT in the polymer backbone. Reproduced with permission.^[^ ^140^ ^]^ Copyright 2024, Wiley‐VCH. c) Regioregular multicomponent conjugated backbones‐based stretchable semiconducting polymer. Reproduced with permission.^[^ ^109^ ^]^ Copyright 2024, Wiley‐VCH.

Oh et al. synthesized a semiconducting polymer through Stille polymerization, incorporating non‐conjugated 2,6‐pyridine dicarboxamide (PDCA) segments into the DPP backbone to introduce hydrogen bonding (**Figure** **11**a).^[^ ^100^ ^]^ Different ratios of PDCA were used to create a series of polymers (P1 to P4) with varying stretchability. Polymers with higher PDCA content (P2, P3, and P4) exhibited lower elastic moduli and higher stretchability compared with the fully conjugated polymer (P1). Specifically, P3 demonstrated excellent mechanical performance, maintaining high field‐effect mobility (over 1 cm^2^ V s^−1^) even under 100% strain; furthermore, its mobility almost fully recovered after strain release. Wang et al. reported the semiconducting polymer DPPTT‐urethane using a novel “regional conjugation” synthetic strategy, integrating oligo‐DPPTT conjugated units with alkyl urethane non‐conjugated units (Figure 11b).^[^ ^140^ ^]^ The synthesis involved a Pd‐catalyzed Stille coupling polymerization to form the DPPTT‐hydroxy polymer, which was then end‐capped with 4‐bromobenzyl alcohol and subsequently polymerized with hexamethylene diisocyanate to yield the final DPPTT‐urethane polymer. Incorporating urethane segments into the polymer backbone significantly influenced both the electrical properties and stretchability of the material, decreasing the overall crystallinity and chain aggregation, thus enhancing stretchability. Consequently, the DPPTT‐urethane stretchable transistors demonstrated high mobility (up to 1.7 cm^2^ V s^−1^) and maintained stable electrical performance even under 100% strain and after 1000 stretch‐release cycles at 25% strain. Chen et al. designed multifunctional integrated polymer semiconductors with intrinsic stretchability and high mobility (Figure 11c).^[^ ^109^ ^]^ They developed pyridal[1,2,3]triazole‐thiophene co‐structured tetrapolymers with full‐backbone coplanarity and significant inter/intramolecular noncovalent interactions, enhancing the short‐range order and stress‐dissipation capabilities of the polymer chains. These polymers exhibited high crystallinity, excellent carrier transport with mobilities exceeding 1 cm^2^ V s^−1^, and controllable near‐infrared luminescence. The regioregular multicomponent conjugated backbones enabled dense packing and a high crack onset strain of over 100%. By employing a homologous blending strategy, they further enhanced the color‐tunable luminescent properties while maintaining the mechanical and electrical properties. This blend system achieved a record quantum yield‐mobility (Φ · μ) of 0.43 cm^2^ V s^−1^, outperforming most reported high‐performance optoelectronic polymer semiconductors.

#### 3.4.2 Side‐Chain Engineering

Side‐chain engineering is another pivotal approach in designing stretchable polymer semiconductors, with the side chains attached to the polymer backbone determining the solubility, processability, and mechanical properties of the polymer. Initially designed to enhance solubility, side chains also significantly impact the polymer chain conformation, solution‐state aggregation, and solid‐state molecular packing. Various side‐chain dependent stretched polymer films have been explored, such as poly(butyl acrylate),^[^ ^141^ ^]^ alkyl chains,^[^ ^142^, ^143^ ^]^ siloxane,^[^ ^144^ ^]^ carbosilane,^[^ ^145^, ^146^, ^147^ ^]^ fluorine,^[^ ^148^, ^149^ ^]^ non‐centrosymmetric units,^[^ ^150^ ^]^ and H‐bonds.^[^ ^151^, ^152^ ^]^ In this section, we introduce representative side chains that affect the strain‐dependent morphology and charge carrier transport properties of intrinsically stretchable transistors.

**Figure 12:**
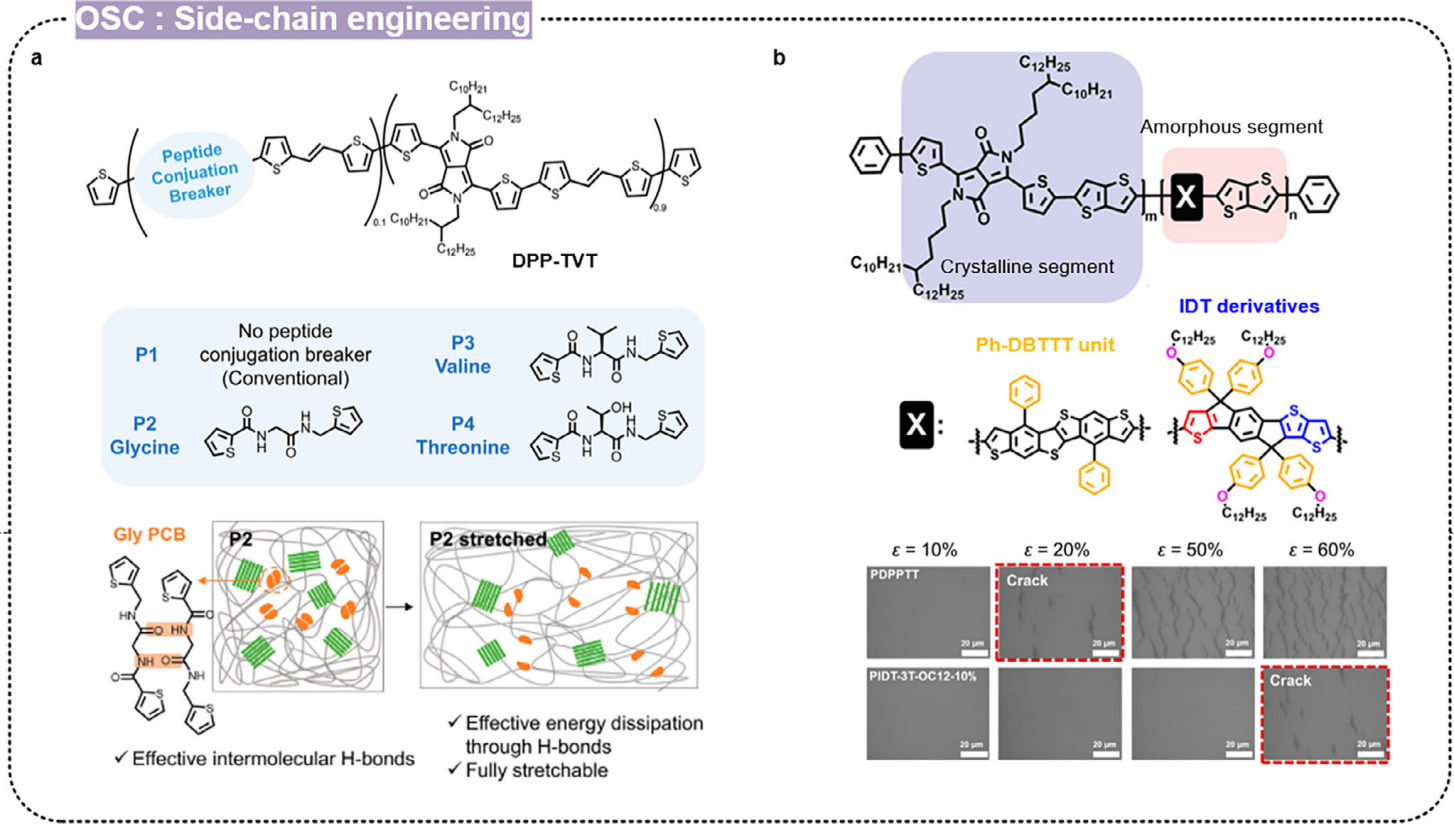
Design strategy for intrinsically stretchable conjugated polymers through side‐chain engineering. a) Peptide conjugation breakers of stretchable organic semiconductors. Reproduced with permission.^[^ ^151^ ^]^ Copyright 2023, American Chemical Society. b) Incorporation of conjugated rigid fused rings with bulky side groups for stretchable polymer semiconductors. Reproduced with permission.^[^ ^65^ ^]^ Copyright 2021, American Chemical Society.

Kwon et al. developed intrinsically stretchable and self‐healing polymer semiconductors by incorporating peptide conjugation breakers (PCBs) into DPP‐based polymers (**Figure** **12**a).^[^ ^151^ ^]^ They synthesized three novel polymers with PCBs based on glycine, valine, and threonine, and investigated the effect of intermolecular and intramolecular hydrogen bonds on the stretchability and self‐healing properties. The polymer containing glycine PCB demonstrated a high mobility of 0.12 cm^2^ V s^−1^ and excellent cyclic durability with a crack‐onset strain exceeding 100%. Furthermore, it maintained mobility even at 100% strain in both rigid and fully stretchable transistors. Liu et al. incorporated conjugated rigid fused rings with bulky side groups while maintaining a conjugated polymer backbone (Figure 12b).^[^ ^65^ ^]^ They synthesized a series of polymers, including benzene‐substituted dibenzothiopheno[6,5‐b:6’,5’‐f]thieno[3,2‐b]thiophene (Ph‐DBTTT) and IDT backbone. The IDT‐based polymer PIDT‐3T‐OC12‐10% demonstrated promising electrical and mechanical properties, with a mobility of 0.27 cm^2^/V s at 75% strain and stable performance after hundreds of stretching cycles at 25% strain.

#### 3.4.3 Elastomer Integration

In addition to chemical structure modifications, integrating other materials, such as highly stretchable insulating elastomers, represents a promising strategy for imparting stretchability to polymer semiconductors. This approach leverages the inherent elasticity of elastomers to provide stable mechanical flexibility, enabling the incorporation of materials with high charge carrier mobility but inherently low elasticity. Embedding these high‐mobility materials within a stretchable elastomeric matrix enables the development of composites that combine excellent electronic performance with robust mechanical stretchability. This approach ensures the retention of high‐charge transport characteristics and enhances the overall durability and resilience of stretchable electronic devices.

**Figure 13:**
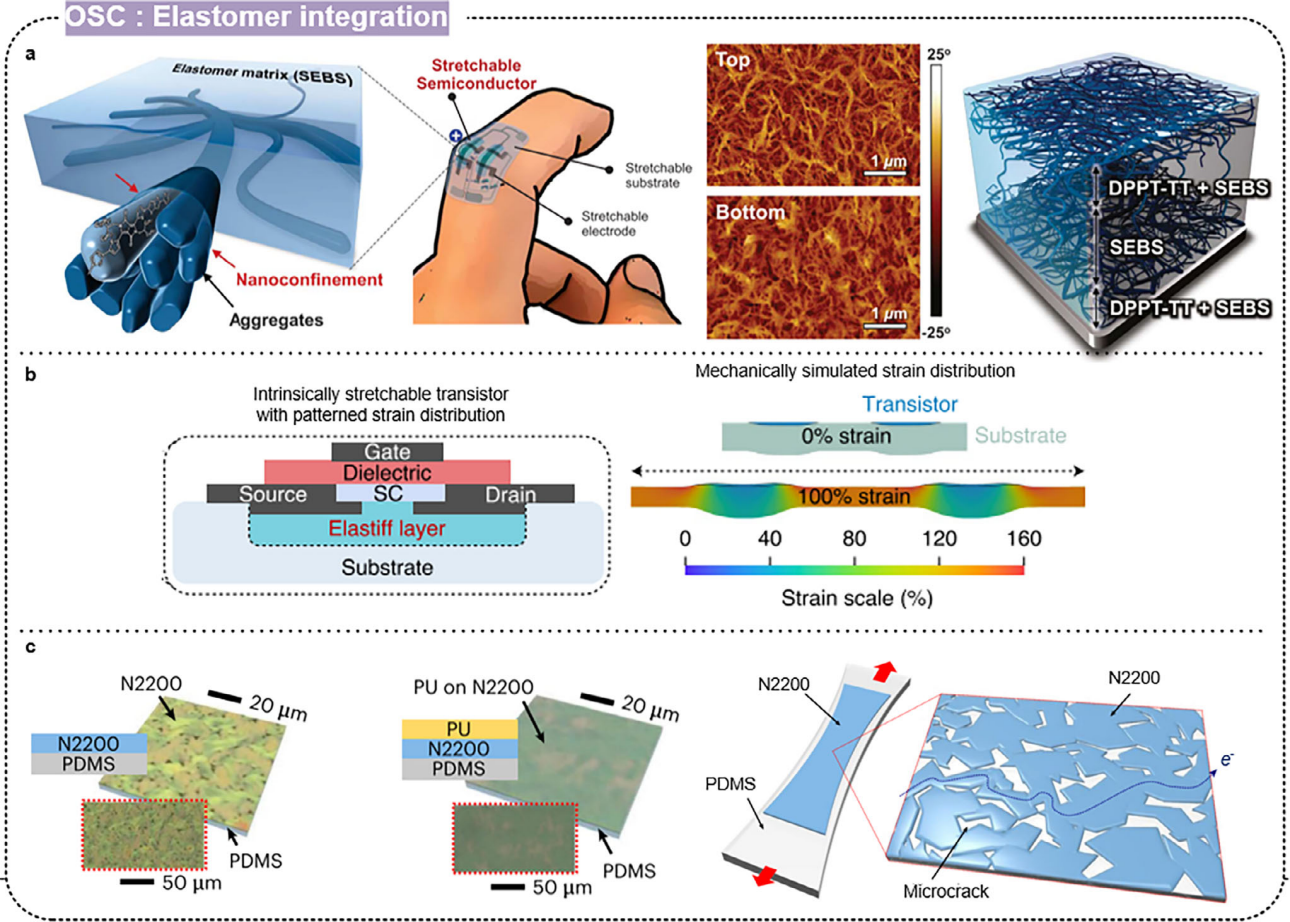
a) Highly stretchable semiconductor polymer based on the nanoconfinement effect. Reproduced with permission.^[^ ^27^ ^]^ Copyright 2017, AAAS. b) Strain‐insensitive transistor with patterned strain distribution. Reproduced with permission.^[^ ^30^ ^]^ Copyright 2021, Springer Nature. c) Elastomer–semiconductor–elastomer stack architecture. Reproduced with permission.^[^ ^105^ ^]^ Copyright 2023, Springer Nature.

Xu et al. reported a nanoconfinement effect in highly stretchable polymer semiconductor films within a soft elastomer matrix (**Figure** **13**a)^[^ ^27^ ^]^ using DPPT‐TT as the high‐mobility semiconducting polymer and SEBS as the elastomer. The nanoconfinement increased the polymer chain dynamics and reduced crystallinity, significantly enhancing the stretchability of the films. The fabricated films withstood strains up to 100% without loss of mobility. The fully stretchable transistors exhibited high biaxial stretchability with minimal change in on‐current, demonstrating excellent mechanical and electrical performance even under extreme deformation. This approach was applied to research on various organic semiconducting polymer‐based stretchable transistors under the name “CONPHINE”.

Wang et al. developed strain‐insensitive, intrinsically stretchable transistor arrays using an all‐elastomer strain engineering process (Figure 13b).^[^ ^30^ ^]^ They introduced patterned regions of mechanical heterogeneity, termed “Elastiff layers,” into the elastomer substrates by selectively varying the cross‐linking density, enabling local stiffening and strain distribution to the active regions of the devices. The resulting transistor arrays exhibited a high device density of 340 transistors per cm^2^ and demonstrated strain insensitivity with less than 5% performance variation when stretched up to 100% strain. Such findings highlight the potential of all‐elastomer fabrication processes for developing high‐density, mechanically robust, and stretchable electronics suitable for monitoring physiological signals and other advanced applications.

Aiming at high performance and mechanical resilience, Shim et al. developed elastic integrated electronics based on a stretchable n‐type elastomer–semiconductor–elastomer (ESE) stack (Figure 13c).^[^ ^105^ ^]^ They used an n‐type organic semiconductor, N2200, encapsulated within a stretchable PU matrix to form a sandwich‐like structure. The encapsulated N2200 maintained a high field‐effect mobility and stable electrical characteristics even under 50% tensile strain and after 3,000 stretching cycles, and long‐term stability over 113 days.

## 4 Fabrication of Stretchable FETs

### 4.1 Deposition Methods

The deposition of the semiconducting layers has been extensively explored to effectively form thin and uniform layers of functional materials, with the deposition methods demanding precise control over film thickness, uniformity, and composition to ensure seamless integration and high device performance. In this section, the different low‐temperature‐based deposition methods of semiconductor layers for stretchable transistors are presented. As shown in **Table** **2**, we explore solution‐based methods such as drop casting, spin coating, dip‐coating, printing technique, and transfer technique, and discuss their advantages and disadvantages in fabricating stretchable devices.

**Table 2:** Summary of deposition methods, with their advantages and disadvantages, and representative examples of semiconducting materials for stretchable FETs.

Deposition methods	Advantages	Disadvantages	Representative Examples
Drop‐casting	‐ Extremely simple process ‐ Low‐cost ‐ No equipment needed	‐ Poor thickness uniformity ‐ Drying defects ‐ Not scalable	CNT^[^ ^80^ ^]^
Spin‐coating	‐ Simple and fast process ‐ High uniformity ‐ Fine thickness control	‐ Material waste ‐ Limited to flat substrates	CdSe/ZnS,^[^ ^74^, ^76^ ^]^ CNT,^[^ ^88^, ^154^ ^]^ P3HT NF,^[^ ^93^ ^]^ MoS_2_,^[^ ^31^ ^]^ PSe‐DPP,^[^ ^55^ ^]^ and CONPHINE^[^ ^28^, ^30^, ^108^ ^]^
Dip‐coating	‐ Simple and low‐cost ‐ Good for alignment	‐ Slow drying process ‐ Sensitive to surface conditions	Te NW^[^ ^72^ ^]^ and CNT^[^ ^33^, ^81^ ^]^
Printing techniques	‐ Compatible with large areas ‐ Scalable production ‐ Patternable deposition	‐ Requires ink optimization (viscosity, surface tension) ‐ Limited resolution for fine structures	CNT,^[^ ^82^, ^86^ ^]^ DPPT‐TT NF,^[^ ^92^ ^]^ Graphene,^[^ ^26^ ^]^ PII2T,^[^ ^101^ ^]^ N2200,^[^ ^105^, ^107^ ^]^ IDTBT,^[^ ^57^ ^]^ and PPT^[^ ^109^ ^]^
Transfer techniques	‐ Enables transfer of high‐quality films ‐ Preserves the performance of brittle materials	‐ Mechanical damage during transfer ‐ Alignment challenges ‐ Limited scalability	CVD grown film (CNT,^[^ ^155^, ^156^ ^]^ SnO_2_ NW,^[^ ^94^ ^]^ graphene,^[^ ^95^, ^96^ ^]^ and MoS_2_^[^ ^98^, ^157^ ^]^) and spin‐coated polymer films^[^ ^27^, ^103^, ^104^ ^]^

The drop casting method affords thin films by directly dropping a semiconductor solution onto a substrate, followed by natural solvent evaporation. While this method is simple and inexpensive to implement, it is only suitable for localized areas or small scales due to the uneven surfaces caused by the viscosity of the solution and the evaporation rate of the solvent. Thus, drop casting, mainly suitable for early‐stage development and proof‐of‐concept demonstrations, may not provide the precision required for high‐performance applications. Liang et al. fabricated SWCNT films by drop‐casting onto a prepared PUA substrate.^[^ ^80^ ^]^ To optimize the performance of FETs, the density of SWCNTs was controlled by varying the amount of the SWCNT ink cast onto the substrate from 5 to 25 tubes per micrometer.

Spin coating is a widely used deposition technique for producing thin, uniform semiconductor films, especially in applications requiring precise film thickness control. In this process, a small amount of solution is dropped onto the center of the substrate upon rotational motion, and the centrifugal force evenly spreads the solution across the substrate. As the rotation continues, the solvent evaporates, forming a thin, uniform film. The main advantage of spin coating lies in its ability to produce uniform and repeatable films while precisely controlling the film thickness. Chen et al. fabricated stretchable polymer semiconductor films through off‐center spin coating.^[^ ^109^ ^]^ Unlike conventional spin‐coating, off‐center spin‐coating involves positioning the substrate slightly away from the center. This slight misalignment enhances film uniformity and reduces surface roughness, leading to higher mobilities in FETs.

Dip‐coating is a simple and cost‐effective method for depositing semiconductor layers, involving immersion of the substrate in a solution containing a semiconductor material, followed by removal at a controlled rate. This technique is often used to align 1D materials such as CNTs in one direction. However, contaminants on the substrate surface can negatively affect the adhesion and uniformity of the deposited layer; therefore, maintaining a clean surface is crucial for obtaining high‐quality thin films. Furthermore, the solution concentration and withdrawal rate significantly influence film thickness and uniformity, requiring careful control. Zhao et al. achieved highly uniform wafer‐scale alignment of TeNWs via dip‐coating at a controlled speed.^[^ ^72^ ^]^ The electrostatic interaction between the positively charged substrate and the negatively charged TeNWs promoted their selective adsorption onto the substrate surface, leading to the formation of a highly uniform, well‐aligned monolayer with a precisely controlled density of 15 to 50 nanowires per micrometer. This alignment, achieved with an angular deviation of ≈ ±6°, was consistent and uniform across the entire wafer—critical for the fabrication of high‐performance, large‐area stretchable electronic devices.

Printing technologies are increasingly employed in semiconductor material deposition because they can print directly on stretchable substrates. Furthermore, they are adaptable, can cover large areas, have high material efficiency, and can pattern complex structures. However, optimizing the viscosity, surface tension, and substrate compatibility of the ink is necessary to avoid uneven film formation, such as the coffee ring effect. Li et al. fabricated an intrinsically stretchable organic electrochemical transistor (OECT) array by multi‐material printing of functional inks.^[^ ^153^ ^]^ To achieve efficient ink deposition, a micro‐structured hydrophilic substrate was developed, with each layer (PEDOT/PSS/Gly ink for the semiconductor, Ag‐20% RTV ink for the electrode, and PAAm ionic hydrogel ink for the dielectric) printed sequentially to fabricate a stretchable OECT array.

Transfer techniques are widely used to integrate high‐quality semiconductor layers onto target substrates. In this process, 1D or 2D semiconductor films are first deposited on a rigid substrate via CVD or thermal evaporation and then transferred to a stretchable target substrate using mechanical or chemical methods. Such techniques offer the advantage of using high‐performance materials that are difficult to deposit directly on stretchable substrates, thereby preserving the electrical properties of the semiconductor layers. However, scalability is a key challenge for transfer methods, as they typically require precise alignment and may be difficult to apply to large‐area or mass‐production conditions. Sun et al. reported an air/liquid interface transfer adherence strategy for stretchable semiconducting films.^[^ ^104^ ^]^ The air‐side transfer process, where the air‐facing side of the film contacts the dielectric layer, avoids the interference of water molecules at the semiconductor‐dielectric interface, thus minimizing the number of adsorbed water molecules, which typically act as traps and reduce carrier mobility. This unique transfer method provides an efficient, damage‐free method for fabricating high‐performance semiconducting films.

In addition to solution‐based strategies, vapor‐phase deposition techniques such as CVD, atomic layer deposition (ALD), thermal evaporation, and sputtering have been extensively employed to fabricate high‐quality semiconductor films, particularly for rigid or flexible electronics. These methods afford dense, uniform, and highly crystalline films with excellent electrical properties; however, they typically require high processing temperatures and produce intrinsically brittle films, which are prone to cracking or delamination under mechanical strain. Therefore, they are generally unsuitable for direct integration in intrinsically stretchable devices without additional structural design, such as serpentine patterning or buckled geometries. While these techniques remain essential for growing or depositing high‐performance 2D materials or oxide films on rigid substrates, their application in stretchable FETs is mostly limited to indirect approaches, such as forming high‐quality films via CVD or evaporation and subsequently transferring them to stretchable substrates through transfer printing or interfacial assembly strategies.

### 4.2 Integration Methods

**Figure 14:**
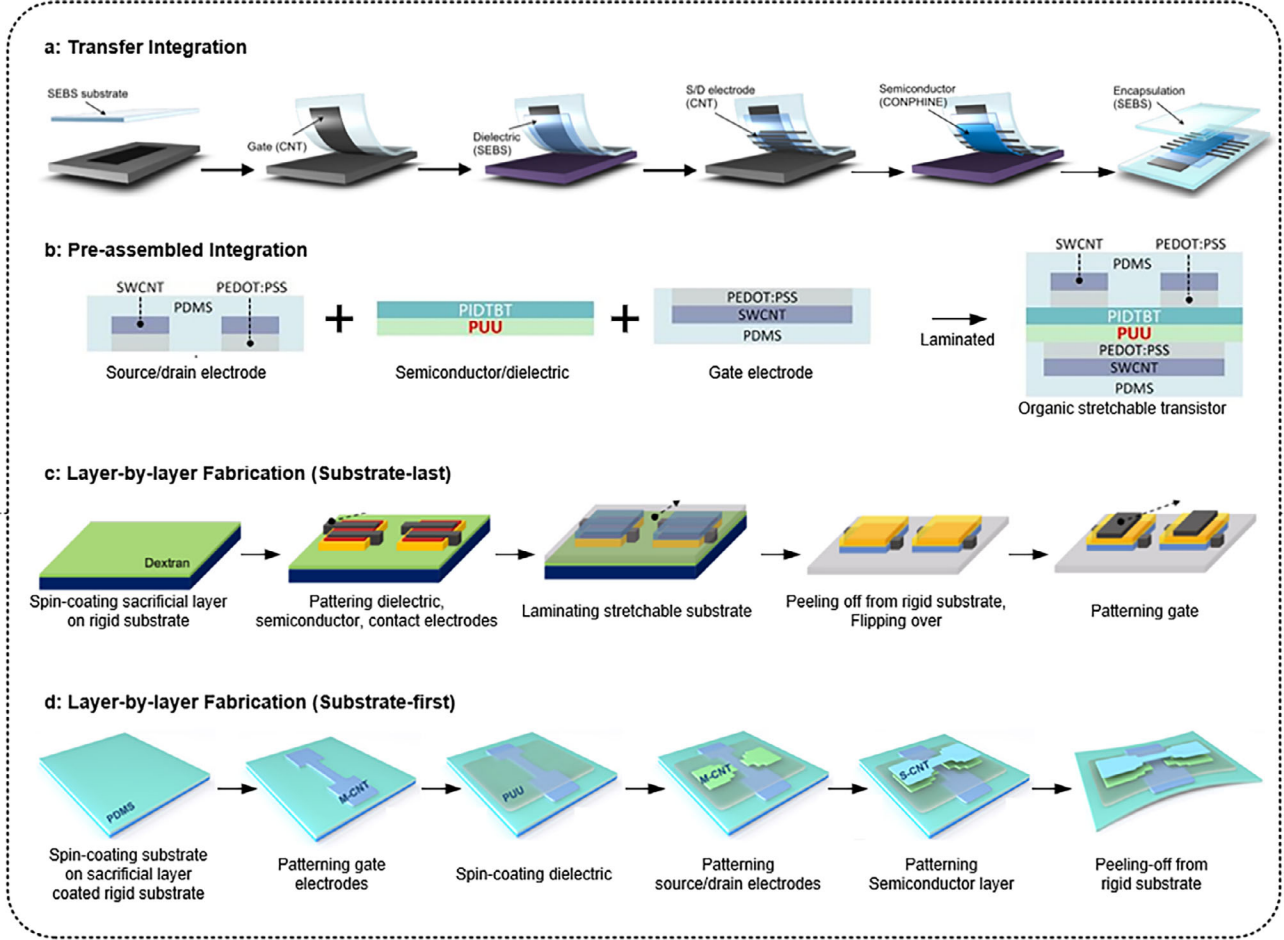
Schematic illustration of the fabrication process for stretchable FETs. a) Transfer integration. Reproduced with permission.^[^ ^27^ ^]^ Copyright 2017, AAAS. b) Pre‐assembled integration. Reproduced with permission.^[^ ^57^ ^]^ Copyright 2024, Wiley‐VCH. c) Layer‐by‐layer fabrication (substrate‐last).^[^ ^30^ ^]^ Copyright 2021, Springer Nature. d) Layer‐by‐layer fabrication (substrate‐first).^[^ ^154^ ^]^ Copyright 2022, Elsevier.

The fabrication of stretchable FETs typically involves sequential integration of multiple layers directly onto the substrate, requiring high‐quality materials with consistent electrical properties and low‐temperature processing to prevent thermal damage during device integration. Depending on the material and processing conditions of each layer, an appropriate integration strategy must be selected. As shown in **Figure** **14**, integration methods for stretchable transistor fabrication are typically categorized into transfer integration, pre‐assembled integration, and layer‐by‐layer fabrication methods (substrate‐last and substrate‐first).

Transfer integration involves sequentially fabricating each device layer on a rigid substrate and subsequently transferring it onto a stretchable substrate (Figure 14a). This method is particularly advantageous for materials requiring high‐temperature processing or complex patterning incompatible with stretchable substrates, allowing the fabrication of high‐quality films and superior device performance. However, challenges include careful handling during transfer, complex layer alignment, susceptibility to mechanical damage, and limited scalability for mass production.

Pre‐assembled integration entails the separate preparation of transistor components into partial stacks, which are then laminated together into the complete transistor device (Figure 14b). This approach facilitates independent optimization of each transistor component and modular manufacturing; however, alignment accuracy during lamination, potential interfacial defects, and complexity in managing the integration flow remain significant challenges.

The layer‐by‐layer fabrication methods include the substrate‐last and substrate‐first approaches. In the substrate‐last method, each transistor layer is sequentially patterned on a sacrificial‐coated rigid substrate, with the stretchable substrate deposited as the final step (Figure 14c). Upon removing the sacrificial layer, the device is released, flipped, and finalized. Conversely, the substrate‐first approach initiates fabrication by depositing the stretchable substrate onto the sacrificial layer‐coated rigid substrate, followed by sequential transistor layer patterning (Figure 14d). Upon completion, the device is released by dissolving the sacrificial layer. Both layer‐by‐layer methods offer precise patterning enabled by rigid substrates; however, subsequent processing is constrained by requirements for orthogonal processing conditions, including limitations due to thermal budgets and solubility compatibility among device layers.

## 5 Applications of Stretchable FETs

Transistors are typically used for signal processing between input and output terminals, being fundamental components in backplane and sensing technologies, and enabling the development of skin‐like electronic devices, wearable electronics, medical implants, and human–machine interfaces. Stretchable FETs have been applied in various devices, including logic and memory devices as well as biomimetic systems. This section presents the main applications of stretchable FETs in various fields.

### 5.1 Sensory Technology

Sensory technology is pivotal in personalized medicine implementation, offering continuous monitoring of the health status of individuals, with extensive applications in human healthcare, human–machine interfaces, intelligent prosthetics, and advanced robotics.^[^ ^115^, ^158^, ^159^ ^]^ E‐skin systems integrate advanced materials and sensory technologies to mimic the sensory functions of human skin, providing real‐time data on environmental changes and physiological conditions. The development of such systems represents a significant advancement in achieving seamless integration between biological and electronic systems, thereby enhancing the capabilities of wearable health monitors, prosthetic devices, and robotic systems.

**Figure 15:**
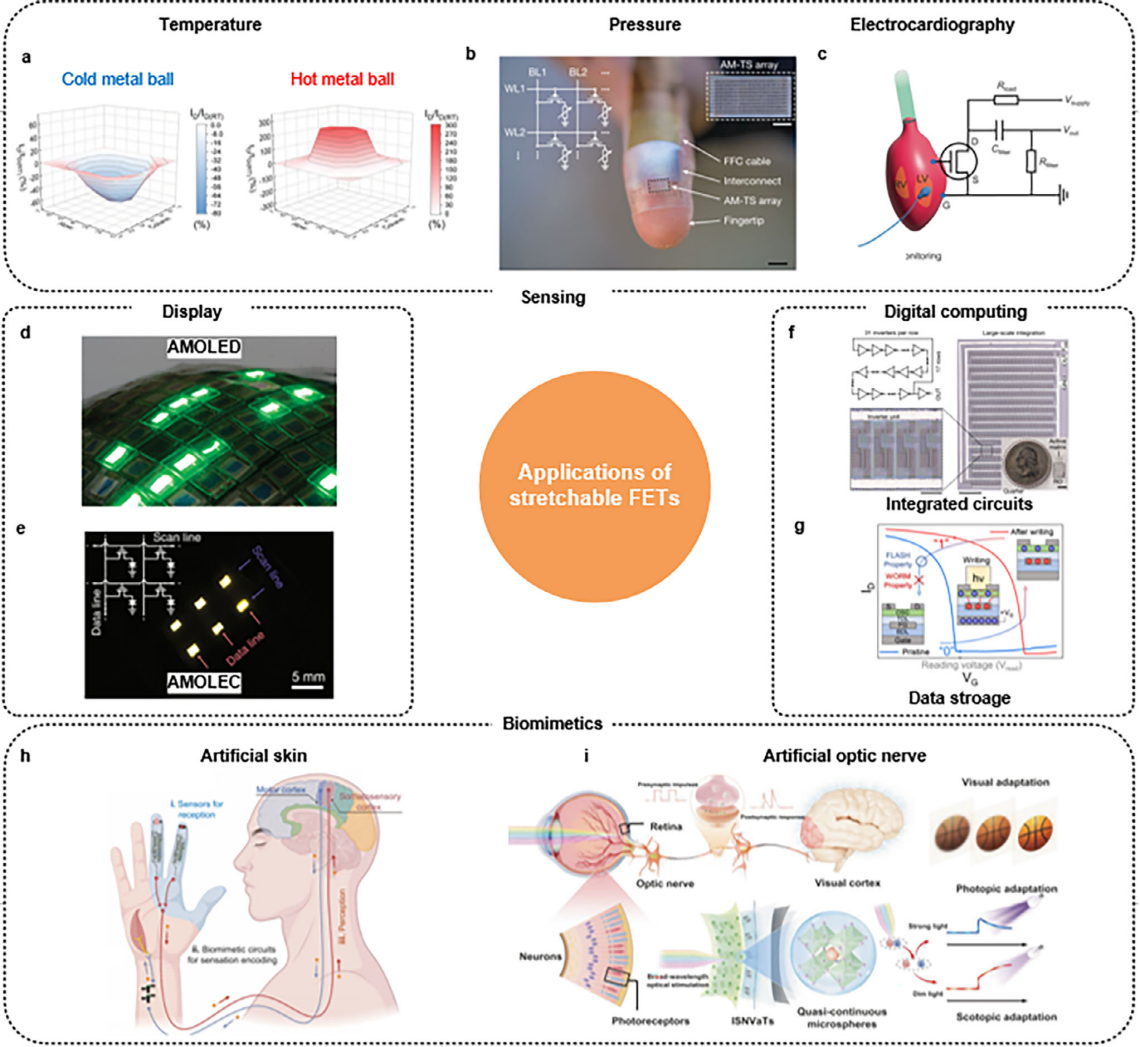
Applications of stretchable FETs. a) Stretchable transistors for active‐matrix temperature sensors. Reproduced with permission.^[^ ^108^ ^]^ Copyright 2023, Wiley‐VCH. b) Stretchable active‐matrix tactile sensors. Reproduced with permission.^[^ ^33^ ^]^ Copyright 2024, Springer Nature. c) OECT sensor for ECG recording on the heart surface and the circuit diagram. Reproduced with permission.^[^ ^167^ ^]^ Copyright 2023, AAAS. d) Stretchable display based on AM‐OLED. Reproduced with permission.^[^ ^171^ ^]^ Copyright 2009, Springer Nature. e) Fully stretchable AMOLEC array. Reproduced with permission.^[^ ^101^ ^]^ Copyright 2020, Springer Nature. f) Intrinsically stretchable and large‐scale integrated circuits. Reproduced with permission.^[^ ^33^ ^]^ Copyright 2024, Springer Nature. g) Intrinsically stretchable floating gate memory transistors. Reproduced with permission.^[^ ^172^ ^]^ Copyright 2024, American Chemical Society. h) Biointegrated E‐skin system for an artificial sensorimotor loop. Reproduced with permission.^[^ ^32^ ^]^ Copyright 2023, AAAS. i) Strain‐insensitive viscoelastic perovskite films for neuromorphic visual adaptation. Reproduced with permission.^[^ ^77^ ^]^ Copyright 2024, Springer Nature.

Kim et al. developed stretchable wearable temperature sensors (**Figure** **15**a)^[^ ^108^ ^]^ by combining organic semiconductor materials and elastic dielectrics, achieving both high performance and mechanical flexibility. Their design includes a stretchable active matrix that maintains functionality under significant strain, enabling real‐time temperature sensing and adaptive responses. The developed transistors detected subtle temperature variations with high precision, making them ideal for integration into skin‐like sensory arrays. The sensors showed high sensitivity with a temperature coefficient of 9.1%/∖ and a fast response time of 0.3 s, making them suitable for healthcare monitoring applications.

Zhong et al. developed a high‐resolution stretchable active‐matrix tactile sensing system (Figure 15b),^[^ ^33^ ^]^ with the optimal material composition and design yielding a robust pressure sensor capable of detecting a wide range of pressures with minimal error margins. The developed sensor exhibited high sensitivity and stability across various pressures and temperatures, with its resistance response decreasing consistently with increasing pressure and temperature. Furthermore, the sensor demonstrated a frequency output change under different pressures, exhibiting reliable and repeatable performance.

Electrocardiogram monitoring is another promising application field for sensors, enabling interpretation of the electrical activity of the heart. OECTs or FETs based on ion gel dielectrics, which have high transconductance and can amplify small signals, have been widely studied.^[^ ^160^, ^161^, ^162^, ^163^, ^164^, ^165^, ^166^ ^]^ Li et al. demonstrated that bio‐adhesive OECTs maintain stable and conformable contact with biological tissues, significantly enhancing signal quality and device performance (Figure 15c).^[^ ^167^ ^]^ Such devices improve signal amplitude and spatial resolution—essential for single‐cell recordings—by maintaining stable contact even during dynamic conditions such as heartbeats and muscle movements. Furthermore, their bio‐adhesive properties simplify the attachment process and avoid cytotoxicity or inflammatory responses.

### 5.2 Display Technology

Over the past 40 years, flat panel displays have significantly advanced, driven by the development of new display modes, enhanced electrical properties of backplane TFTs, and optimized materials and manufacturing processes.^[^ ^168^, ^169^, ^170^ ^]^ However, despite the commercialization of semiconducting materials like low‐temperature polycrystalline silicon (LTPS) and indium‐gallium‐zinc‐oxide (IGZO) TFTs, their application in stretchable wearable devices remains challenging.

Sekitani et al. developed a stretchable active‐matrix organic light‐emitting diode (OLED) display (Figure 15d)^[^ ^171^ ^]^ using highly conductive SWNTs with a fluorinated rubber matrix and achieving high conductivity (>100 S cm^−1^) and stretchability (> 100%). The stretchable display integrated organic transistors and OLEDs, forming a 16 × 16‐pixel active matrix that maintained functionality even when stretched by 30–50%, folded, or crumpled. Liu et al. developed a fully stretchable active‐matrix organic light‐emitting electrochemical cell (OLEC) array, highly promising for developing skin‐like displays (Figure 15e).^[^ ^101^ ^]^ They integrated a stretchable TFT array with stretchable OLECs for a robust, monolithic device via solution processing, by providing the necessary current density to drive the OLEC pixels and ensuring high brightness and stability under mechanical deformation.

### 5.3 Digital Computing

Digital computing involves electronic devices and systems that process information in a discrete binary form, utilizing sequences of 0 and 1s. This foundational technology powers modern computing by performing operations on discrete data through logic gates and circuits, enabling complex computations and data manipulation. Central processing units (CPUs) execute software instructions, while various forms of memory store data and programs. Digital computing encompasses a wide array of applications, from personal and mobile devices to data centers, embedded systems, scientific computing, and artificial intelligence.

Zhong et al. developed intrinsically stretchable, high‐speed, and large‐scale integrated circuits (Figure 15f)^[^ ^33^ ^]^ featuring high‐density transistors with a device density of 100,000 transistors/cm^2^. They achieved a high electrical performance under strain, highlighting the development of a 527‐stage ring oscillator with 1,056 transistors and achieving a stage‐switching frequency greater than 1 MHz. Furthermore, the circuits showed excellent mechanical robustness, maintaining functionality under 100% strain. Such advancements underscore the potential of these circuits for various applications in on‐skin electronics, physiological monitoring, and human–machine interfaces.

The field of digital computing includes memory devices for data storage. Nam et al. reported intrinsically stretchable floating gate memory transistors (Figure 15g),^[^ ^172^ ^]^ achieving high memory on/off ratios greater than 10^4^ and memory windows exceeding 10 V. These devices maintained stable operation across a temperature range from −30 to 60 °C and under different ambient atmospheres such as air, nitrogen, and vacuum. Furthermore, they demonstrated excellent mechanical durability, withstanding up to 50% uniaxial and 30% biaxial strains, and remained functional after being submerged in water for 60 min. The stretchable memory transistors also retained their performance during various deformations when attached to human skin.

### 5.4 Biomimetics

The term biomimetics derives from the Greek words “bio” (life) and “mimetic” (imitation). Biomimetics seeks to address complex human challenges by emulating functions and mechanisms observed in nature, fostering innovation in fields such as engineering, materials science, and technology. Recently, the development of neuromorphic systems that replicate brain‐associated capabilities in learning, memory, and pattern recognition has garnered significant attention. Achieving highly efficient neuromorphic sensory systems requires the creation of artificial synapses characterized by low power consumption and high‐density integration. Recent studies have concentrated on incorporating artificial synapses into brain‐like systems to replicate the functionalities of biomimetic sensory and motor nervous systems.^[^ ^76^, ^173^, ^174^, ^175^, ^176^ ^]^ These artificial synapses respond to stimuli such as light and pressure, and have been integrated with external sensors to emulate somatosensory, visual, auditory, gustatory, and olfactory nerve pathways, thereby forming a comprehensive sensory nervous system.

Wang et al. developed a monolithic, soft E‐skin system that emulates the sensory feedback functions of biological skin (Figure 15h).^[^ ^32^ ^]^ The system integrates various sensors to mimic biological receptors, employing low‐voltage‐driven circuits to encode the sensor signals into pulse trains, similar to biological sensory systems. Stretchable artificial synapses actuate muscle movements in response to these signals, mimicking the natural sensorimotor loop. Using a trilayer high‐permittivity elastomeric dielectric ensured low operation voltage, low power consumption, and high circuit integration, while maintaining good tissue conformability and mechanical softness for biointegration. Tested on a live rat model, the E‐skin successfully emulated the sensory feedback loop, triggering muscle contractions in response to pressure stimuli without causing skin irritation.

Wang et al. developed an intrinsically stretchable neuromorphic vision‐adaptive transistor (ISNVaT) using a defect‐tunable viscoelastic perovskite film (Figure 15i).^[^ ^77^ ^]^ This film mimics the adaptive and sensory functions of biological systems by integrating high photosensitivity and mechanical flexibility. The perovskite QDs are dispersed in an elastomeric matrix to create a quasi‐continuous microsphere morphology, ensuring both stretchability and efficient photoelectric performance. This structure enables ISNVaT to achieve ultra‐low energy consumption, a high paired‐pulse facilitation (PPF) index, and rapid adaptive imaging capabilities comparable to human vision.

## 6 Summary and Outlook

Stretchable transistors for electronic skin applications have significantly advanced the development of high‐performance, highly stretchable, and stable electronic devices using various semiconductor nanomaterials. This review highlights the major challenges and promising development trends in stretchable semiconductor materials, focusing on the balance between electrical performance and stretchability, and emphasizing the potential for improvement of each material. The growing demand for wearable devices has increased the need for efficient, convenient, and high‐performance stretchable transistors. To fully unlock the potential of this field, in‐depth research is needed in several key areas:

### 6.1 Material Innovation

Several substrates, electrodes, dielectrics, and semiconductor materials have been developed for realizing stretchable transistors. Despite the substantial progress in stretchable substrates and electrodes with satisfactory performance, developing novel dielectric materials and semiconductors remains essential for commercialization and must continue to advance. QDs offer excellent optical and electronic properties (e.g., tunable band gap and high quantum yield); however, their stability, toxicity, scalability, and integration require further improvement for commercialization, particularly in displays, solar cells, and stretchable devices. CNTs have garnered significant attention as a promising 1D semiconductor material for stretchable electronics. Their high carrier mobility (≈20 cm^2^ V s^−1^) and ability to enable high‐performance, high‐density stretchable displays offer the potential for commercializing electronic skin applications. Strategies that improve the alignment of CNTs and reduce junction resistance have enhanced their electrical properties; however, achieving isotropic stretchability remains a challenge. 2D materials also exhibit promise as stretchable semiconductors, owing to their atomic‐layer‐thin structures, high carrier mobility, and mechanical flexibility. However, graphene lacks a bandgap, leading to low on/off current ratios, thus requiring further research into effective doping techniques. Meanwhile, TMDCs face limitations related to high processing temperatures and scalability for large‐area applications. OSCs are actively studied for use in stretchable FETs, owing to their inherent mechanical plasticity, compatibility with solution‐based low‐temperature processes, and their ability to be integrated with stretchable dielectric materials. However, despite improvements in stretchability, manufacturing yield, and device density, OSCs still suffer from relatively lower electrical performance. In addition to electrical and mechanical requirements, future material innovation must also prioritize environmental sustainability and biocompatibility, particularly for wearable and implantable devices. Incorporating eco‐friendly semiconductors based on biodegradable polymers or naturally derived materials could reduce long‐term environmental impact, while biocompatible interfaces ensure safer interaction with human skin or tissues. In summary, developing novel stretchable semiconductor materials with superior electrical properties at low processing temperatures is essential. Therefore, new approaches, along with process innovations to achieve stretchability and high‐performance stretchable materials, are required to overcome the limitations of current stretchable semiconductor materials.

### 6.2 Device Fabrication and Integration

The fabrication and integration of intrinsically stretchable FETs pose significant challenges that differ from those of conventional rigid or flexible electronics. In particular, the thermally sensitive nature of stretchable substrates necessitates low‐temperature processing. It is important to achieve high‐quality, uniform films in low‐temperature based process. In addition to deposition techniques, patterning and alignment on stretchable substrates require significant adaptation. Traditional photolithography, must be replaced or modified to ensure high‐resolution and alignment precision on stretchable devices. At the integration level, surface engineering becomes critical. For example, chemical surface treatments can enhance surface energy, improve ink wettability, and promote layer adhesion. Physical texturing or nanostructuring of surfaces can increase interfacial contact area, enabling better mechanical anchoring and minimizing delamination under strain. Interface engineering, particularly at semiconductor‐dielectric and semiconductor‐contact junctions, plays a central role in maintaining electrical performance during mechanical deformation. Soft interlayers, gradient mechanical modulus designs, or self‐assembled monolayers (SAMs) can reduce interfacial strain and prevent mechanical mismatch‐induced failure. In particular, integrating conductive polymers or compliant metal composites at the contact regions can maintain charge injection efficiency while accommodating stretch. Encapsulation strategies, using stretchable barrier layers like PDMS or parylene, also enhance mechanical durability and environmental stability without sacrificing stretchability. For scalable manufacturing, future stretchable electronics must move beyond proof‐of‐concept devices and be compatible with roll‐to‐roll, inkjet printing, or screen‐printing processes on large‐area substrates. However, ensuring process reproducibility and layer alignment over large areas under mechanical strain remains a key limitation. To this end, modular assembly approaches, in which pre‐fabricated stretchable circuit units are integrated via soft interconnects, or transfer printing techniques, which allow precise placement of active materials on arbitrary substrates, offer promising pathways. Additionally, structural engineering, such as using serpentine designs, kirigami cuts, or wrinkled architectures, can further enhance mechanical robustness and cyclic strain endurance. In summary, addressing the multifaceted challenges of surface and interface compatibility, mechanical stability, and scalable processing is essential for the commercial realization of stretchable FETs. Future research should explore chemically tailored interfaces, mechanically adaptive interlayers, and integrated patterning schemes that enable high‐throughput, low‐cost production of stretchable logic, sensing, and computing systems.

### 6.3 Applications

Stretchable FETs are incredibly promising, with their application expected to expand across diverse fields such as personalized medicine, smart wearable devices, and biomimetics. Their integration into E‐skin systems will continue to revolutionize healthcare monitoring, enabling more accurate and continuous tracking of vital signs, environmental stimuli, and user movements. Furthermore, advancements in stretchable display technologies will pave the way for flexible, skin‐like screens for consumer electronics, providing new opportunities for personalized, adaptable user interfaces. In digital computing, the development of highly stretchable integrated circuits and memory devices will enable more robust and resilient flexible electronics, facilitating the emergence of wearable, portable computing systems. Furthermore, as neuromorphic systems and artificial intelligence evolve, stretchable FETs will play a critical role in mimicking biological sensory functions. Their mechanical biocompatibility and potential for multimodal integration make them ideal candidates for next‐generation artificial synapses, neuron‐inspired logic gates, and distributed sensor networks in neuromorphic systems. Beyond conventional computing, these devices can support adaptive learning and closed‐loop feedback when integrated with soft robotics or neural interfaces, offering transformative capabilities in prosthetics, brain–machine interfaces, and wearable neuroelectronics. However, key challenges remain, including improving the long‐term durability, scalability, and electrical performance of these stretchable devices, particularly under repeated mechanical strain. Future research should focus on overcoming these challenges by developing novel materials, enhancing device integration, and ensuring the reliable operation of stretchable FETs.

In conclusion, the development of stretchable FETs marks a transformative leap in the field of stretchable electronics, offering significant potential for applications in wearable devices, biomimetic systems, and soft robotics. Although substantial progress has been made in material design, deposition techniques, and device integration, challenges persist in achieving scalable production, long‐term reliability, and high‐performance devices that maintain their electrical properties under mechanical deformation. The choice of materials for each layer—semiconductors, dielectrics, and electrodes—as well as the optimization of low‐temperature fabrication processes and precise patterning techniques, is crucial for overcoming these obstacles. Future research should focus on enhancing the mechanical stability and environmental resilience of these devices while also ensuring their efficient and cost‐effective large‐scale fabrication. With continued research and development, stretchable FETs are poised to revolutionize various sectors, from healthcare monitoring and biomimetic systems to next‐generation electronics and human–machine interfaces, providing the foundation for a new era of highly integrated, stretchable, and adaptable electronic devices.

## Conflict of Interest

The authors declare no conflict of interest.
